# A genome-wide identification and comparative analysis of the lentil *MLO* genes

**DOI:** 10.1371/journal.pone.0194945

**Published:** 2018-03-23

**Authors:** Carlos Polanco, Luis E. Sáenz de Miera, Kirstin Bett, Marcelino Pérez de la Vega

**Affiliations:** 1 Area de Genética, Departamento de Biología Molecular, Universidad de León, León, Spain; 2 Department of Plant Sciences, University of Saskatchewan, Saskatoon, Saskatchewan, Canada; Università Politecnica delle Marche, ITALY

## Abstract

Powdery mildew is a widespread fungal plant disease that can cause significant losses in many crops. Some *MLO* genes (*Mildew resistance locus O*) have proved to confer a durable resistance to powdery mildew in several species. Resistance granted by the *MLO* gene family members has prompted an increasing interest in characterizing these genes and implementing their use in plant breeding. Lentil (*Lens culinaris* Medik.) is a widely grown food legume almost exclusively consumed as dry seed with an average world production of 4.5 million tons. Powdery mildew causes severe losses on certain lentil cultivars under particular environmental conditions. Data mining of the lentil CDC Redberry draft genome allowed to identify up to 15 gene sequences with homology to known *MLO* genes, designated as *LcMLOs*. Further characterization of these gene sequences and their deduced protein sequences demonstrated conformity with key MLO protein characteristics such as the presence of transmembrane and calmodulin binding domains, as well as that of other conserved motifs. Phylogenetic and other comparative analyses revealed that *LcMLO1* and *LcMLO3* are the most likely gene orthologs related to powdery mildew response in other species, sharing a high similarity with other known resistance genes of dicot species, such as pea *PsMLO1* and *Medicago truncatula MtMLO1* and *MtMLO3*. Sets of primers were designed as tools to PCR amplify the genomic sequences of *LcMLO1* and *LcMLO3*, also to screen lentil germplasm in search of resistance mutants. Primers were used to obtain the complete sequences of these two genes in all of the six wild lentil relatives. Respective to each gene, all *Lens* sequences shared a high similarity. Likewise, we used these primers to screen a working collection of 58 cultivated and 23 wild lentil accessions in search of length polymorphisms present in these two genes. All these data widen the insights on this gene family and can be useful for breeding programs in lentil and close related species.

## Introduction

Powdery mildew is an airborne plant disease widespread in temperate climates that is caused by ascomycete fungi of the order Erysiphales and that can cause significant harvest losses in field-crops, fruit crops, and ornamental plants. The *Mildew resistance locus O* (*MLO)* gene family is a subject of intense research because some of its members have proved to confer a durable resistance to powdery mildew in several crop species. *MLO* genes are present as small families in the genomes of all higher plant species, both monocots and dicots [1] and the described number of genes ranges from 8 to 39 [2]. Powdery mildew resistant *mlo* mutants were first described in barley (*Hordeum vulgare*) [3] and were characterized later in the model plant *Arabidopsis thaliana* [4]. Since then, *mlo*-based resistance has been described, in chronological order, in the following plant species: tomato (*Solanum lycopersicum*), pea (*Pisum sativum*), strawberry (*Fragaria vesca*), pepper (*Capsicum annuum*), bread wheat (*Triticum aestivum*), cucumber (*Cucumis sativus*), rose (*Rosa hybrida*), tobacco (*Nicotiana tabacum*), melon (*Cucumis melo*), grapevine (*Vitis vinifera*), and apple (*Malus domestica*), as reviewed by Kusch and Panstruga [5]. Loss-of-function mutant alleles of the *MLO* gene confer a broad-spectrum resistance to almost all known isolates of the barley powdery mildew pathogen, *Blumeria graminis* f.sp. *hordei*, and they seem to also confer resistance to the corresponding fungal pathogen species in other plants species [2, 5–6]. Loss-of-function *mlo* alleles are caused by nucleotide substitutions and indels, large deletions or aberrant splicing in different plant species [7–10]. Undesirable pleiotropic effects of the *mlo* mutants, such as a premature senescence or a reduced plant size, have been described in some species but have not been observed in others [5]. MLO proteins are located at the plasma membrane and contain several transmembrane domains but their function remains uncertain, although some of them are related to leaf senescence, morphological development and stress responses [11]. MLO proteins have been phylogenetically grouped into seven clades (I to VII) [2]. Monocot MLO proteins known to be associated with powdery mildew susceptibility belong to clade IV while in dicot species they sort into clade V [2, 12].

Other genes associated to the *mlo* resistance response exist (e.g, *Ror1* and *Ror2* in barley), which seem to belong to the superfamily of *SNARE* (Soluble N-ethylmalemide-sensitive factor Attachment protein REceptor) genes [13]. SNAREs are small proteins often inserted into membranes that can function on either target membranes (t-SNAREs) or transport vesicles (v-SNAREs) [14]. Vesicle-associated membrane proteins or VAMPs (also referred to as synaptobrevins), are the transport vesicle residing SNARE proteins involved in the membrane fusion processes between transport vesicles and target membranes [15]. Extracellular immune responses to ascomycete and oomycete pathogens in *Arabidopsis* are dependent on vesicle-associated secretion mediated by the SNARE proteins PEN1 (syntaxin), SNAP33 and endomembrane-resident VAMP721/722 [16].

The durable resistance conferred by *MLO* gene family members in crop species has prompted an increasing interest regarding these genes and their use in plant breeding. From a breeding point of view, the first step is to identify and characterize the *MLO* genes present in a particular crop species followed by the development of breeding tools, such as genetic markers, for their use in crop improvement. This interest is highlighted by the set of recently-published papers devoted to the identification and characterization of these genes and/or validation of their associated markers: *Solanum* species [17], pigeon pea and common bean [18], *Vitis flexuosa* [19], legume species [20], pea [21–22], cotton [23], tomato [24], cucumber [25], and chick pea [26].

Lentil (*Lens culinaris* Medik. subsp. *culinaris*) is one of the earliest domesticated plant species in the Fertile Crescent. It is a diploid (2n = 14), self-pollinating, annual cool season grain legume that has a relatively large genome size (~4 Gb). It is normally grown in temperate semi-arid regions, usually in rotation with cereals and plays an important role in human nutrition, as a source of energy, proteins and iron. In addition, they are an important dietary source of fiber, minerals vitamins and antioxidants. Likewise, lentils contribute to improved soil quality by replenishing the soil nitrogen levels. The crop is now extensively cultivated throughout Western Asia, Northern Africa, the Indian subcontinent, Australia and North America, in particular in Canada, the current leader in global production and a major exporter [27–28].

In lentil, powdery mildew is caused by *Erysiphe trifolii*. It can cause severe losses in some lentil cultivars under certain environmental conditions, thus posing a great threat for lentil production. Powdery mildew affects all the above ground parts of the lentil plant including leaves, stems and pods [29–30]. Pea (*Pisum sativum* L.) is the closest cultivated species to lentil (both are included in the Vicieae tribe) in which the genetics of resistance to powdery mildew has been studied. In pea, three loci for resistance to powdery mildew, caused by *Erysiphe pisi* Boerema & Verh., named *er1 (PsMLO1)*, *er2* and *Er3* have been described. Gene *er1* has been widely used in pea breeding programs and provides complete or incomplete resistance under field conditions at different locations. Resistance conferred by this gene has proved to be durable. The expression of *er2* is influenced by temperature and leaf age. Gene *Er3* was identified in *P*. *fulvum* and introduced into *P*. *sativum* crop germoplasm by sexual crosses [10, 31].

Wide crosses with wild relative species, even of the secondary and tertiary gene pools, have widely been used to increase the genetic variability of crop species or for the introgression of desirable genes such as resistance genes. Wild lentil relatives have been broadly used to analyze quantitative loci (QTLs) related to yield and other plant characters as well as a source of resistance genes to both biotic and abiotic stresses [32–37]. Sources of resistance to powdery mildew have been identified in wild lentils [35] but the inheritance patterns have not been subjected to genetic analysis yet.

The first aim of this work was to identify and characterize the members of the *MLO* gene family in lentil and identify those orthologous to the *MLO* genes known to confer resistance to powdery mildew in other crop species. These were then used to design primers which can be used in the screening of germplasm collections in the search of putative resistant lines. The final aim was to test these primers in a small collection of lentil germplasm. Complementarily, we have identified and characterized the orthologous of genes which are known to interact with *MLO* genes in the response to powdery mildew in other plant species. The results will prove useful for screening lentil germplasm (wild and cultivated) in search of putative resistance mutants, trace introgressions during backcrossing of resistance genes, and for other breeding purposes.

## Materials and methods

### Identification and validation of the lentil *MLO* genes

A Blastn search of the lentil genome draft (“CDC Redberry” cv., pre-release v0.8 [38]) allowed us to identify a large and non-annotated scaffold containing the whole lentil gene homologous to pea *PsMLO1*. Other tBlastn searches using soybean [39] and *Medicago* [20] MLO protein sequences as query were carried out to identify additional scaffolds containing the homologous lentil genes. Coding DNA regions (CDS) were annotated using the GeneWise tool [40]. The deduced lentil protein sequences were validated by reciprocal BLAST searches carried out on the protein NCBI datasets of the *M*. *truncatula*, chickpea, and common bean genomes. The *MLO* annotations in the lentil draft genome assembly v1.2 can be accessed through the lentil JBrowse or BLAST function at http://knowpulse.usask.ca.

### Protein characterization and motif prediction

The deduced amino acid sequences of the putative *LcMLO* genes were analyzed by several prediction programs to identify functional domains, to determine protein topologies, and sub-cellular localizations [41–47]. The prediction programs were run with default settings of the online servers listed in S1 Table. The CDS sequences of the annotated *MLO* genes in lentil were aligned with the available sequences of other legumes to construct a phylogenetic tree using MEGA7: Molecular Evolutionary Genetic Analysis version 7.0 software [48].

Phylogenetic trees were constructed by adding the lentil and *Glycine* MLO sequences [39] to the legume sequences previously used in a MLO gene analysis [20].

### Sequence amplifications by PCR of lentil *MLO1* and *MLO3*

Genomic DNA was extracted from 15-day-old seedling leaves using the DNeasy Plant Mini Kit (Qiagen Corp., Santa Clarita, CA) following the manufacturer’s protocol. The materials included 58 cultivated lentil accessions and cultivars (*Lens culinaris* Medik. subsp. *culinaris*) and four accessions of the wild *L*. *culinaris* subsp. *orientalis* (Boiss.) Ponert (the wild ancestor of the cultivated lentil), six accessions of *L*. *odemensis* (Godr.) Ladiz., four accessions of *L*. *nigricans* (Bieb.) Godr., and three accessions each of *L*. *tomentosus* Ladiz., *L*. *ervoides* (Bring.) Grande, and *L*. *lamottei* Czfr (S2 Table).

For *LcMLO1* a set of seven primer pairs (A to G) were initially used to obtain the genomic sequences of the subspecies *culinaris* and *orientalis* (S3 Table). Another five pairs (A2, A3, C2, F2, and G2) were needed in order to complete all of the sequences belonging to the wild species. In the same way, five primer pairs (A-M3 to E-M3) were initially used for *LcMLO3*, which were complemented with three other pairs (A2-M3, C2-M3, and E2-M3) (S3 Table).

PCR reactions were carried out in 25 μL reaction volume including ca. 100 ng of DNA, 0.5 μM of region specific forward and reverse primers (S3 Table), PCR buffer (10 mM Tris-HCl; 50 mM KCl;0.1% Triton X-100; pH 9.0), MgCl (2.5 mM), dNTPs (2.5 mM each), and 1 U of *Taq* polymerase. The PCR program started with a 15-min initial activation step at 95°C followed by 15 cycles of denaturation at 94°C for 30 s, annealing at 58°C for 90 s and extension at 72°C for 60 s, and 25 cycles of denaturation at 94°C for 30 s, annealing at 52° C for 90 s and extension at 72°C for 60 s. The program ended with a final extension at 60°C for 15 min. Amplifications were performed in Applied Biosystems 2700 thermal cyclers. Amplified products were electrophoresed in 1.5% agarose gels and visualized by ethidium bromide staining.

A full set of PCR products from at least one accession of each species and subspecies was sequenced directly twice, using the Sanger method and capillary electrophoresis at the sequencing facility of the University of León (Spain), and then assembled for both *MLO1* and *MLO3* genes (S2 Table).

## Results

### Identification and organization of the lentil MLOs

**Fig 1:**
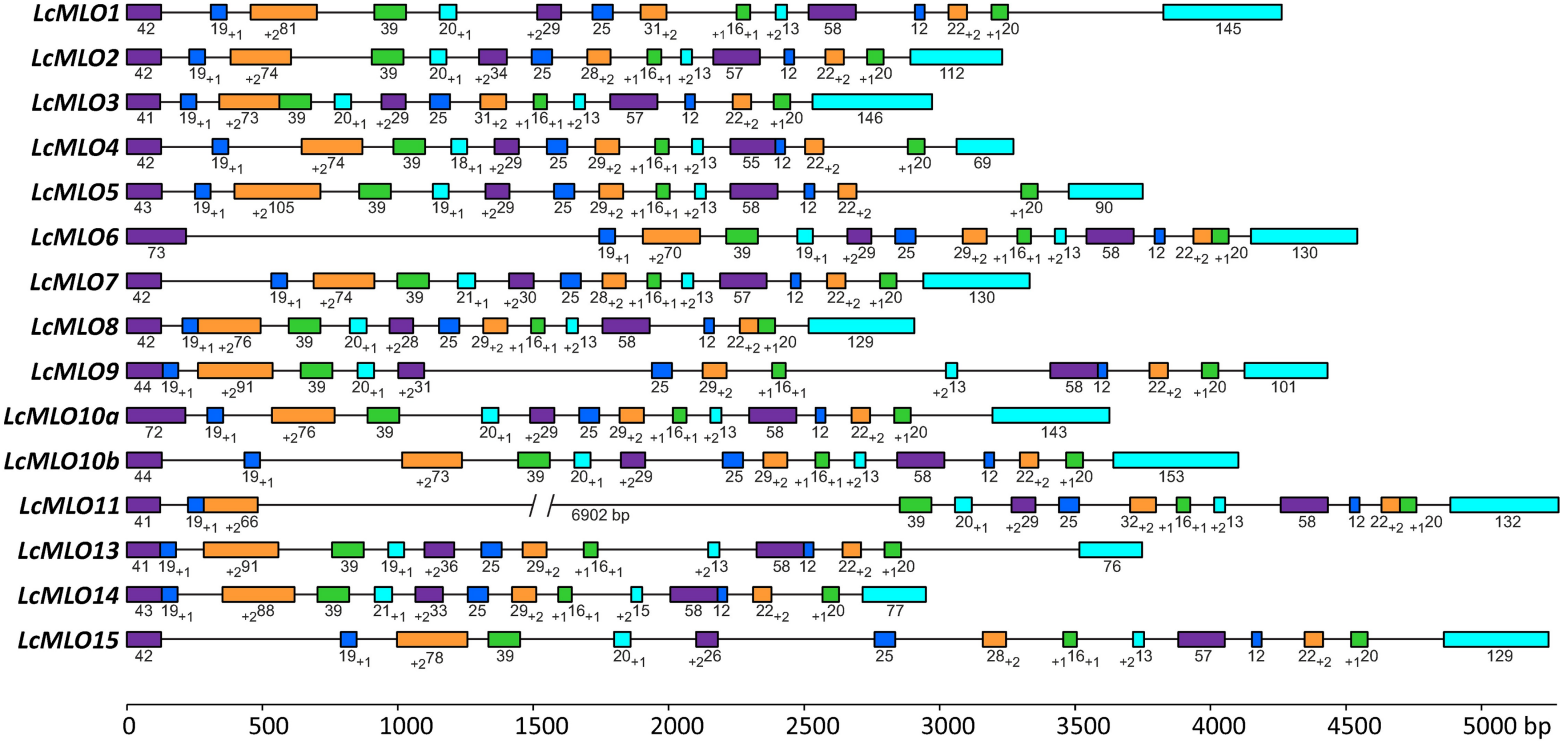
Intron-exon organization of 15 *LcMLO* genes from the CDC Redberry lentil genome. Exons are depicted as rectangles and introns as lines. The exon color code depicts sequence conservation. The numbers below of exons indicate the number of complete triplets and the extra nucleotides (subscripts) in each exon to maintain the exon phasing. A double bar is used to indicate the schematic shortening of the long intron of *LcMLO11* (i.e., not drawn to scale).

Data mining of the lentil (*Lens culinaris* Medik.) CDC Redberry draft genome identified 15 unique sequences with homology to *M*. *truncatula* and *A*. *thaliana MLO* genes (Fig 1; S4 Table). The lentil genes were named as *LcMLO* and numbered according the nomenclature in *M*. *truncatula* [20], so that the pairs from *Medicago* and lentil sharing the highest similarity carry the same serial number (*MtMLO1* and *LcMLO1*, for instance). In addition, eight sequences predicted to encode truncated proteins were also identified. These shorter sequences were considered pseudogenes and named according to the *M*. *truncatula* gene designation following the same procedure (e.g., *ψLcMLO9* shared the highest similarity with *MtMLO9*). Lentil pseudogenes were not included in further phylogenetic analyses.

**Fig 2:**
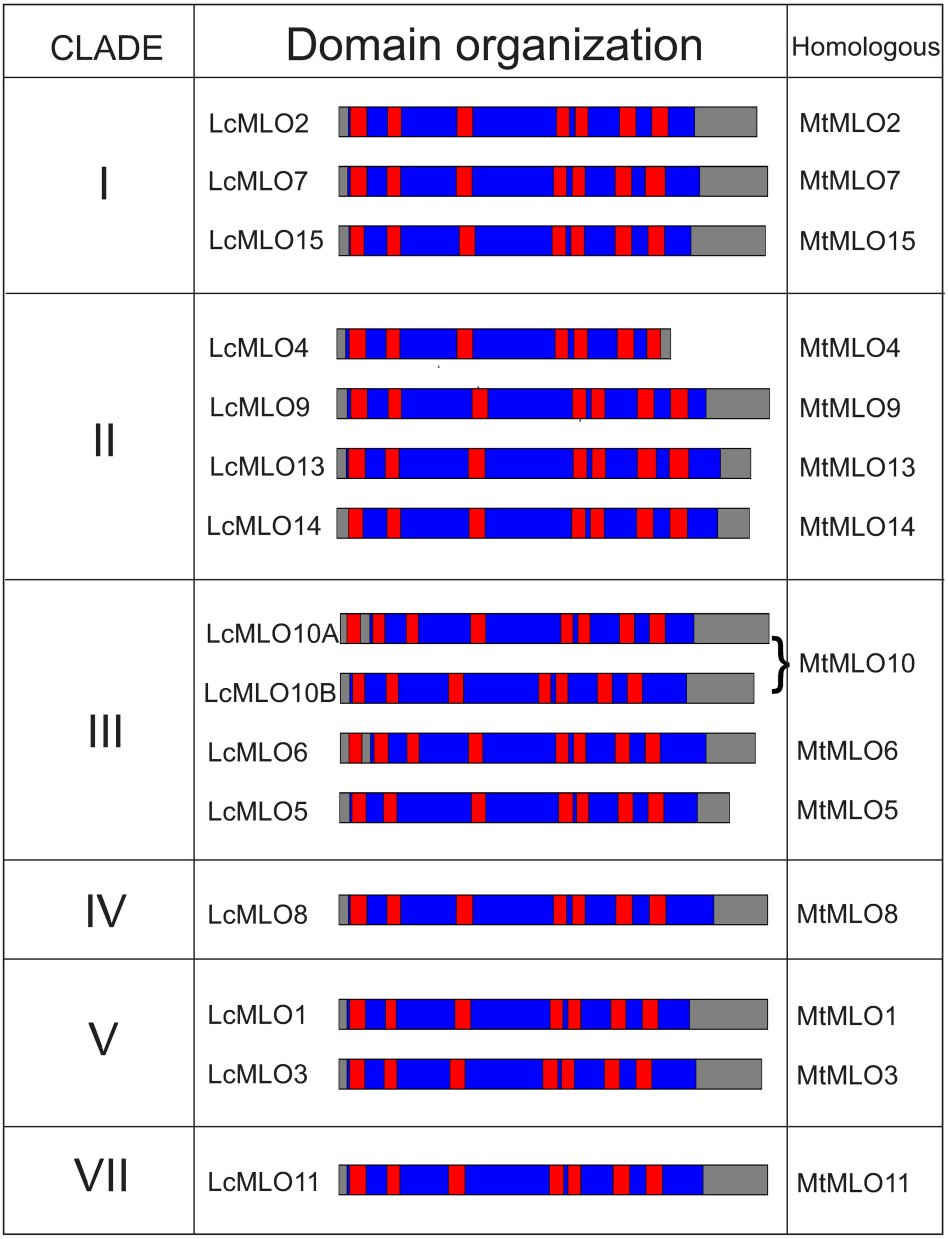
Domain organization of the 15 deduced MLO protein sequences of lentil. Red boxes denote the location of the transmembrane (TM) regions predicted using the CCTOP consensus method. The blue areas denote the MLO domain. LcMLO sequences are grouped according to the legume *MLO* phylogenetic tree clades. *M*. *truncatula* homolog proteins named after Rispail and Rubiales [20].

The 15 lentil MLO candidates were not only similar in sequence to previously described MLO proteins, but also shared characteristic features of these proteins such as presence of both transmembrane (TM) and calmodulin binding domains (CaMBD) (Fig 2, [1]). All the *LcMLO* genes were multi-exonic containing 13 to 15 exons per sequence (Fig 1). Transcript lengths from the start to the stop codons ranged from 2,904 nt (*LcMLO8*) to 9,813 nt (*LcMLO11*, characterized by the presence of a large intron) (Fig 1; S4 Table) and lengths of the CDSs varied from 1,461 nt (LCMLO4) to 1,794 nt (*LcMLO10A*) (S4 Table). The in-silico deduced lentil protein sequences ranged from 487 to 598 amino acids. Exon-intron border positions were further verified by comparing the genomic sequences with transcripts of the lentil cultivar Alpo that were assembled using RNAseq reads from other lentil studies in progress (data not shown).

**Fig 3:**
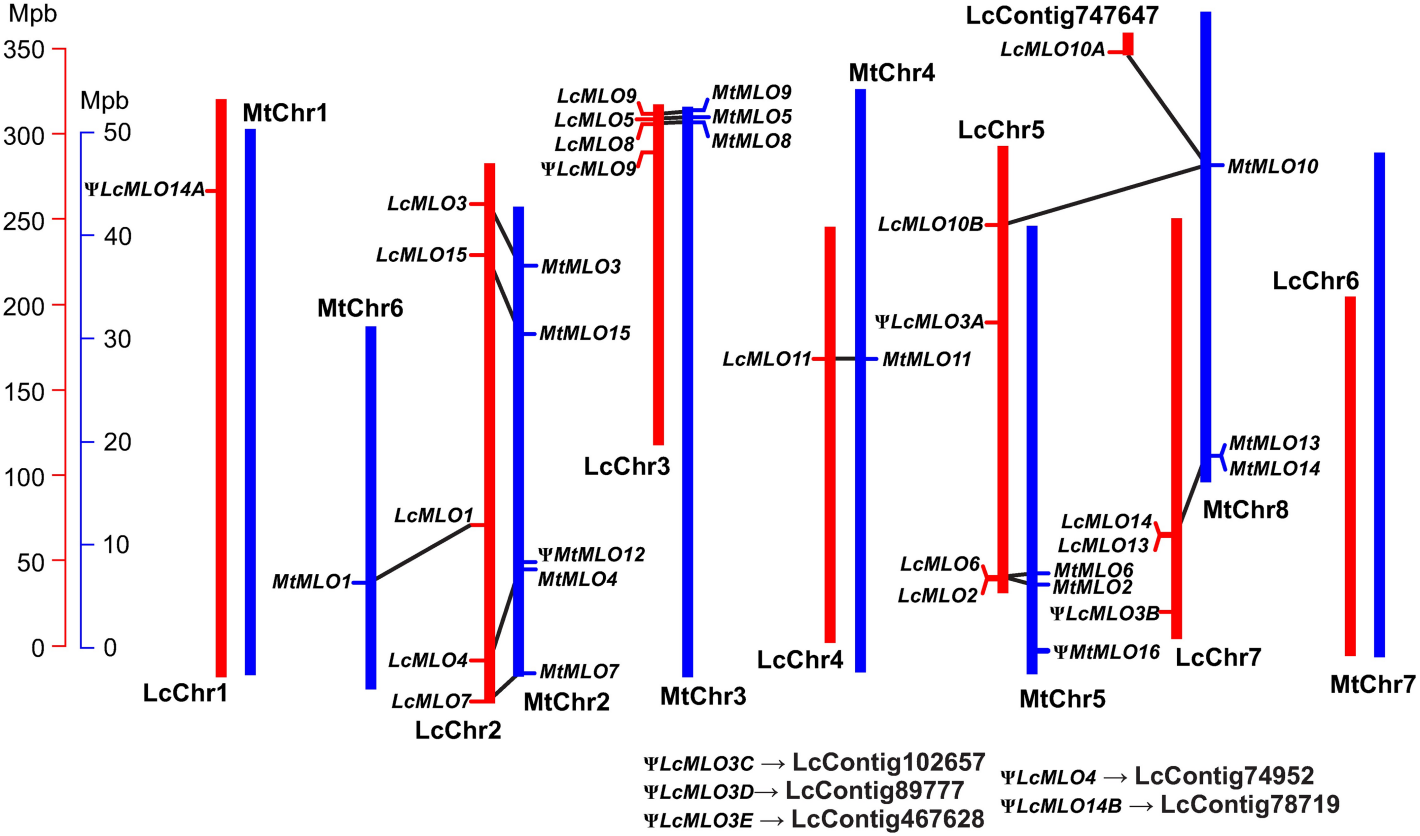
Location of the *LcMLO* loci and comparison with the corresponding *MtMLO*. The physical map of lentil CDC Redberry genome assembly v1.2 is represented by red vertical lines; the *M*. *truncatula* v4.1 physical map by blue vertical lines. Black lines link the homologs genes.

The *LcMLO* sequences were localized on five pseudomolecules of the more recent lentil genome assembly v1.2 (http://knowpulse.usask.ca) corresponding to the chromosome set of this species. They shared a high level of synteny when compared with the location of the *MtMLO* genes in *M*. *truncatula* genome (Fig 3), with the putative lentil ortholog of each *MtMLO* gene occupying syntenic positions in the physical map. For instance, four genes keep the same relative positions in chromosome 2 of lentil (LcChr2) and MtChr2; the cluster of three genes in distal positions on chromosomes LcChr3 and MtChr3; or the pair *MtMLO13* and *MtMLO14* and the pair *ψLcMLO13* and *LcMLO14* tightly linked on MtChr8 and LcChr7, respectively. *LcMLO1* could be implicated in the chromosome rearrangement LcChr2 vs. MtChr2-Chr6. On the other hand, two sequences, *LcMLO10A* and *LcMLO10B*, closely similar to *MtMLO10* were identified in an unassigned contig (LcContig747647) and on LcChr5, respectively. No putative orthologs to the truncated sequences *MtMLO12* and *MtMLO16* were found in the lentil assembly.

#### Characterization of proteins and domain organization

MLO proteins are characterized by the presence of seven TM domains and a MLO functional domain [20]. To determine whether the *LcMLO* genes shared these characteristics, their deduced amino acid sequences were analyzed by means of a set of prediction software used previously in the characterization of legume MLOs [20]. The 15 sequences were predicted to contain a single MLO domain (Fig 2). Different online tools, such as TMHMM or InterProScan [42, 45], use different algorithms to predict TM domains, and lead to differences in the number of predicted TMs in some LcMLO sequences (S5 Table). To select the final number of TMs of each sequence we used the CCTOP (Constrained Consensus TOPology) prediction server which localizes membrane spanning regions and the orientation of segments between them as a consensus of 10 different methods [43]. Seven TMs were predicted in 13 LcMLOs while eight were predicted in LcMLO6 and LcMLO10A. No signal peptide was predicted in the LcMLOs. The search for GO terms identified all LcMLO as integral component of membranes; in addition, LcMLO1 and LcMLO4 were also included in the ion transport biological process category.

**Fig 4:**
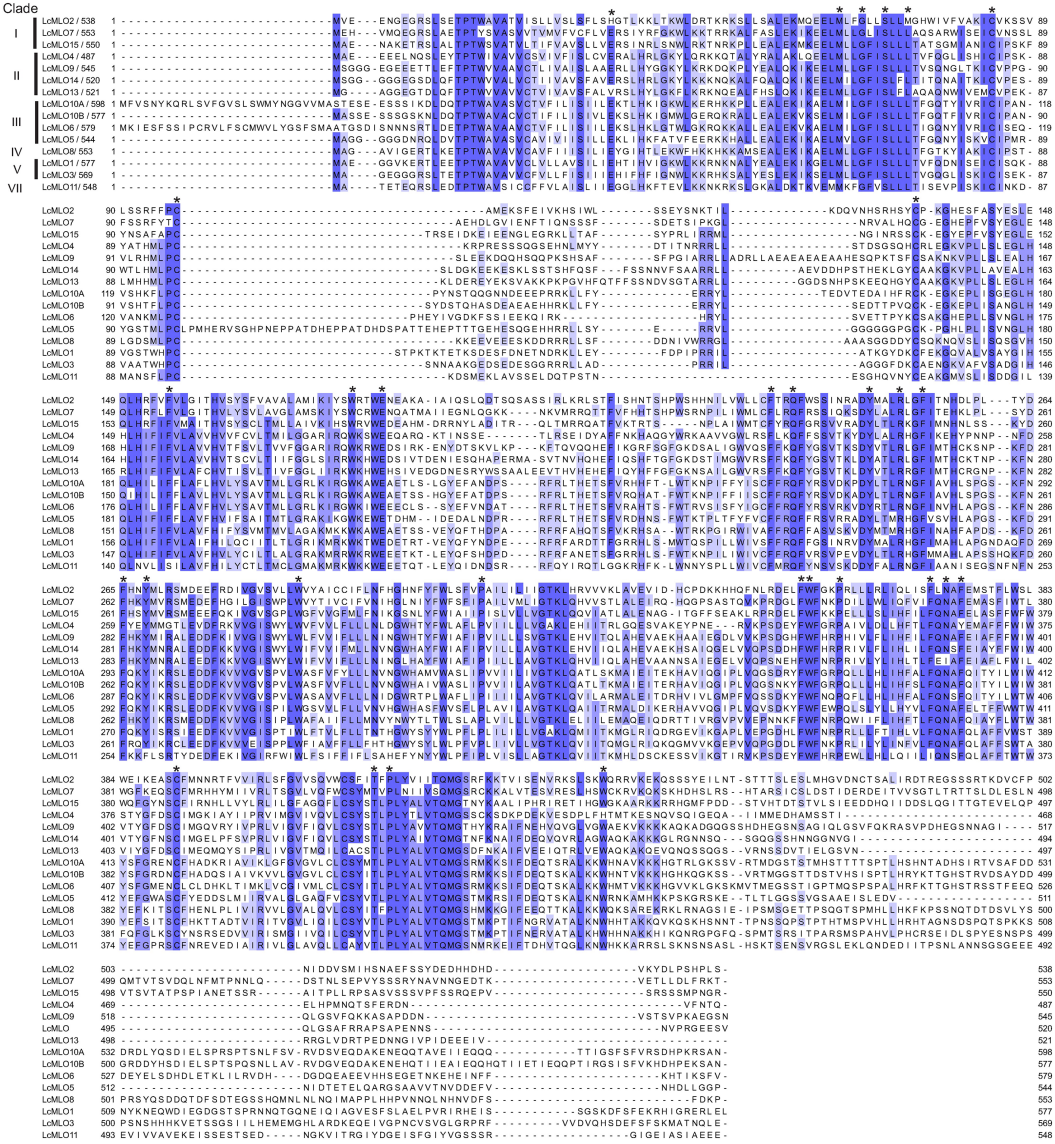
Alignment of the 15 LcMLO protein sequences. Blue shading and its intensity indicate conserved sites and the degree of conservation. Sequences are arranged according to their phylogenetic clade. Asterisks denote the 30 conserved residues along the MLO proteins in higher plants [45].

Conserved domains and invariable amino acids have been described among MLOs of higher plants (monocots and dicots) [1, 49]. The invariable 30 amino acid residues previously described [49] were identified in the LcMLOs; most of them are present in all of the 15 sequences and only eight positions showed amino acid substitutions in individual sequences (Fig 4, S6 Table). Likewise, bioinformatic analyses identified calmodulin binding domains in the intracellular C-end of all LcMLO sequences.

#### Phylogenetic analysis

**Fig 5:**
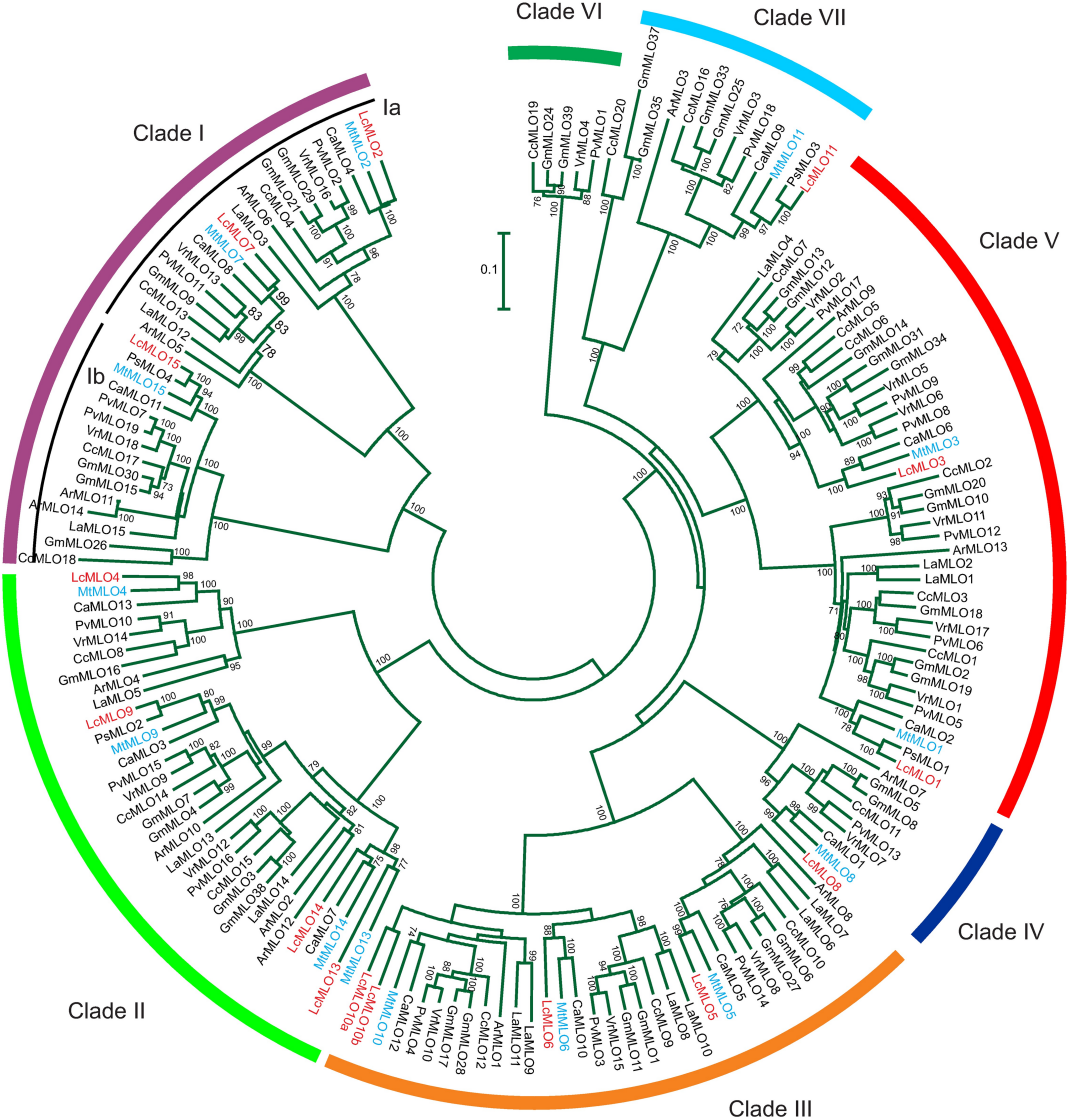
Neighbor-Joining phylogenetic tree of the legume *MLO* genes. Nucleotide CDS sequences and the Kimura two-parameter genetic distance were used. Clades and subclades were designated according Acevedo-Garcia et al. [2] and Rispail and Rubiales [20]. Boot-strap node support values lower than 70 have been omitted. *Lens culinaris* and *Medicago truncatula* genes are represented in red and blue colors, respectively. Other species are abbreviated as follow: Ar, *Arachis* spp.; Ca, *Cicer arietinum*; Cc; *Cajanus cajan*; Gm, *Glycine max*; La, *Lupinus angustifolius*; Ps, *Pisum sativum*; Pv, *Phaseolus vulgaris*; Vg, *Vigna radiata*. Sequences of legume species other than those from *Lens* obtained from previous studies [20, 39].

The *MLO* family was previously subdivided in seven well-supported gene clades including two subclades within clade I [20]. The lentil coding DNA sequences (CDS) were aligned and compared with those homologs derived from previously studied legume species [20]: *Pisum sativum* (pea), *Medicago truncatula* (barrel medic), *Cicer arietinum* (chickpea), *Lupinus angustifolius* (narrow-leaved lupin), *Phaseolus vulgaris* (common bean), *Arachis* spp. (groundnut), *Cajanus cajan* (pigeon pea) and *Vigna radiata* (mung bean), and with 35 *Glycine max* (soybean) sequences [39]. Pea and lentil are in the tribe Vicieae, while chickpea and *Medicago* are included in sister tribes close to the genus *Lens* (Cicereae and Trifolieae, respectively), and all these tribes are included in the Galegoid species clade and are also known as cool season legumes. Soybean, common bean, mung bean and pigeon pea are included in the Phaseoloid clade, narrow-leaved lupin in the Genistoid, and groundnuts in the Dalbergioid clade [50]. A first phylogenetic tree was obtained using the Tamura two-parameter distances and Neighbor-Joining (Fig 5). The lentil sequences were distributed among six of the seven previously defined clades [2, 20]. Clade VI does not include any sequences of the above named cool season legumes, the closest taxa to *Lens*. The *LcMLO1 and LcMLO3* sequences were included in clade V, where all of the known *MLO* genes associated with powdery mildew resistance in dicots are found (Fig 5). *LcMLO1*, *PsMLO1* (the gene responsible of powdery mildew resistance in pea) and *MtMLO1* clustered within a subgroup of Clade V, while *LcMLO3* and *MtMLO3* positioned in another subgroup. The lentil *LcMLO8* gene was the only one included in group IV, which contains the barley powdery mildew resistance genes [2]. The trees obtained with the maximum likelihood and maximum parsimony algorithms showed similar topologies (not shown).

#### Further characterization of the *Lens MLO1* and *MLO3 genes*

**Fig 6:**
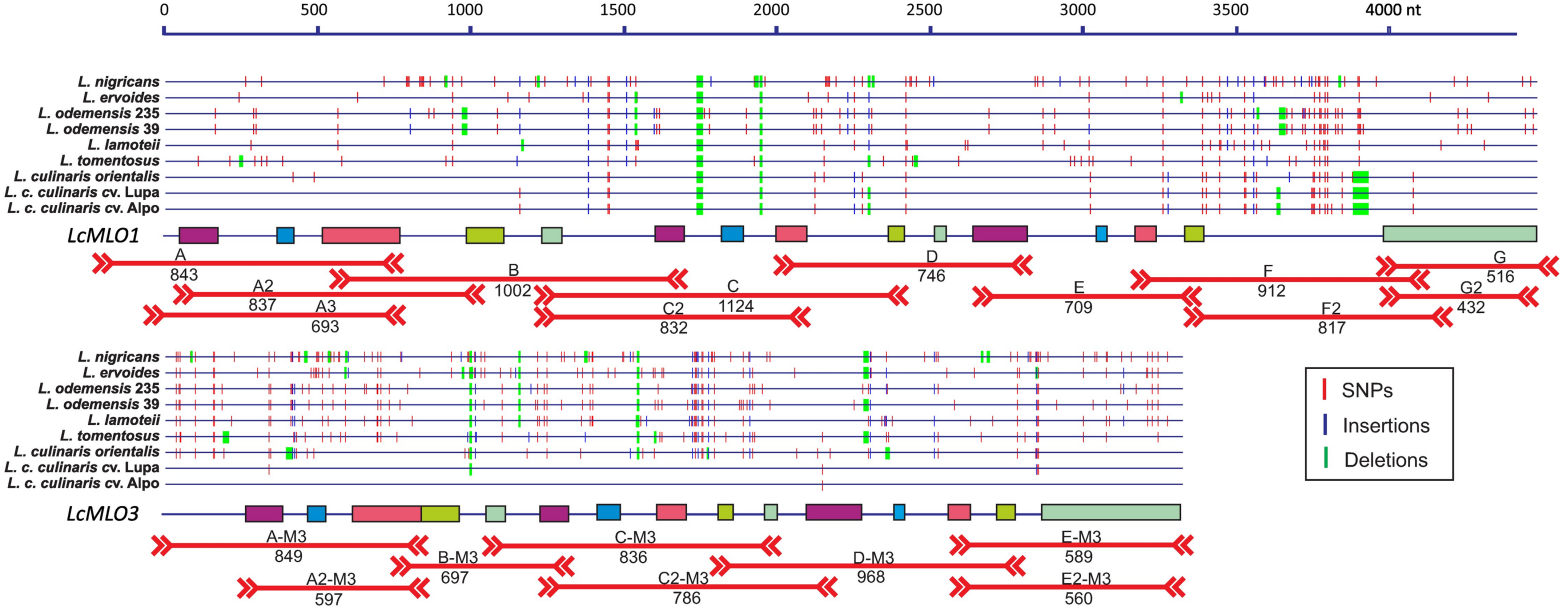
Intron-exon organization of *Lens LcMLO1* and *LcMLO3* genes. Vertical colored bars indicate the location of SNPs (red), insertions (purple) and deletions (green) along of the genes of the species sequenced listed to the left. PCR-amplified regions are indicated with orange horizontal lines showing the corresponding primer-pair name on top. The numbers under the orange lines indicate the expected amplicon size (bp) of the “CDC Redberry” lentil cultivar.

The *LcMLO1* and *LcMLO3* genes were selected for further analyses, as they are the most likely candidates to be involved in the response to powdery mildew in lentil due to their high similarity with known genes involved in this kind of response, and because of their grouping in clade V which contains known powdery mildew susceptibility genes in other species. Two PCR primer sets were designed to amplify each of the *LcMLO1* and *LcMLO3* sequences. The primer annealing site locations and the amplification strategy are depicted in Fig 6.

Both *MLO1* and *MLO3* gene sequences from all *Lens* species were similar within each set of homologs, with most of the sequence differences clustering in introns (Fig 6). The *MLO1* transcript sequences from the start codon to the stop codon ranged from the 4,217 nt of *L*. *culinaris* to the 4,283 nt of *L*. *ervoides*. The exception was *L*. *nigricans* (4,521 nt), the largest *L*. *nigricans* sequence being due to a differentially larger intron. The CDS sequence in all *Lens* species was 1,734 nt. The *MLO3* transcripts ranged from 2,970 nt in *L*. *culinaris* to 3,025 nt in *L*. *tomentosus*. The *MLO3* CDS sequences showed two slightly different lengths: 1,710 nt and 1,719 nt. The main difference was due to the insertion of three amino acids (GVA) in relation to the *L*. *culinaris* sequences observed in three accessions: *L*. *lamotei*, *L*. *ervoides* and one of the two *L*. *odemensis* sampled. Considering the deduced protein sequences, the total number of polymorphic sites observed between all *MLO1* sequences was eight (all substitutions), while for *MLO3*, substitutions in 24 positions and two indels (three and one amino acid) were observed (Fig 6, S1 and S2 Figs). The amino acid sequences of LcMLO1 and LcMLO3 indicating the locations of the TM and calmodulin domains are depicted in S1 and S2 Figs, respectively. The phylogenetic relationships among the *MLO1* and *MLO3* genomic sequences of the analyzed *Lens* accessions are depicted in S3 Fig.

The same primer sets (Fig 6; S3 Table) were also used to test the amplification in a working collection of 58 cultivated and 23 wild accessions of lentil (S2 Table). The corresponding partial gene regions were PCR amplified in all of the accessions detecting no length polymorphisms among the lentil materials analyzed within each of the *Lens* species or subspecies.

#### Characterization of lentil gene sequences related to MLO function

Blast searches using the barley ROR2 protein sequence on the Leguminosae subset of the NCBI database identified a series of sequences with values between 2 e^-106^ and 6 e^-101^. These proteins are designated as syntaxin 121, or 122 in the case of *M*. *trundatula*. A further search using the UniProt database confirmed the identity of the *Medicago* sequence, with a 100% identity, as MTR_1g056550 (Syntaxin of plants 122 protein). This *Medicago* sequence was used to identify sequences found in the transcriptomes of lentil cv. Cassab [51], cv. Alpo, and *L*. *odemensis* ILL235 (unpublished data). The *L*. *odemensis* sequence differed by a single amino acid from the *L*. *culinaris* sequences; the deduced protein sequence of both *Lens* species (named Syntaxin 122) consists of 335 amino acids, slightly longer than the barley ROR2 protein with 318 amino acids. S4A Fig shows the phylogenetic tree obtained with ROR2, using the legume sequences and the *Arabidopsis* homolog. In *Medicago* and *Cajanus* additional similar sequences were detected. As can be observed in S5 Fig, legume sequences shared a high similarity in the central part of the protein, which is relatively conserved with *Arabidopsis* and even with the barley ROR2.

The *Arabidopsis* VAMP721 and 722 sequences were used to search for similar sequences in the above mentioned transcriptomes of *Lens* and also in the NCBI databases to retrieve sequences of other species. Several putative VAMP sequences were identified in *Lens* (S4B Fig). Two of them, one derived from *culinaris* while the other from *odemensis*, clustered with the *Arabidopsis* VAMP sequences, although the bootstrap supporting value was low. The deduced lengths of the named VAMP721/722 proteins of *Lens* were 220 (*L*. *odemensis*) and 221 (cv. Alpo) amino acids. All the VAMP-like proteins of *Lens* shared functional characteristics with the *Arabidopsis* VAMP 721 and 722, as will be discussed further on.

The same procedure as for VAMP proteins was followed with the *Arabidopsis* SNAP33 sequence. In this case, a single sequence per legume genus was identified except for *Lens*, in which two sequences per species were identified (S4C Fig). *Lens* species SNAP33-1 and SNAP33-2 were 366 and 298 amino acids long, respectively, and they included two SNARE domains. Sequence analysis by software at online servers indicated no other protein characteristics or domains.

## Discussion

The *MLO* gene family is important in crop breeding because some gene alleles confer a durable resistance to powdery mildew [2, 7, 17, 25]. Apart from the well documented role of some *MLOs* in the response to powdery mildew, little is known about the function of these genes. *MLO* gene mutations can modulate premature leaf senescence processes, root thigmomorphogenesis, pollen functioning, or stress responses [11, 12].

Mining the lentil draft genome assembly has allowed us to identify and characterize 15 *LcMLO* sequences (Fig 1; S4 Table). This number falls within of the functional *MLO* gene range described in the majority of plant species analyzed so far, including legume species in which the number ranges from 13 to 20 [2, 20]. In addition to a high sequence similarity, the lentil MLO deduced protein sequences shared key characteristics with other known MLOs, such as the presence of seven transmembrane domains, a calmodulin binding domain and the absence of leader peptide (Fig 2; S5 Table). Shorter sequences, most likely pseudogenes, were also found in lentil genome, although any of them were identified as orthologs of the *MtMLO12* and *MtMLO16* psudogenes described in *M*. *truncatula* [20]. This kind of truncated sequences has previously been described in other legume species [20, 26].

The Lc*MLO* genes and pseudogenes were distributed in six of the seven lentil chromosomes (pseudomolecules) and an unassigned contig (Fig 3). This wide distribution throughout of the genome is also characteristic of the other legume genomes [20]. Another characteristic shared with the other legume species in that some Lc*MLO* genes are grouped in a low-number of clusters. The comparison between *LcMLO* chromosome locations and the *Medicago* orthologs has indicated a high degree of synteny (Fig 3). This result agrees with the high level of micro- and macro-synteny that exists for this gene family among the legume genomes [20, 52, 53]. Rispail and Rubiales [20] pointed out that segmental and tandem duplications, these latter with a minor role, have played a key part with regard to the genomic distribution of the *MLO* genes in legumes. *LcMLO13* and *LcMLO14* are likely examples of a tandem duplication; they are located adjacent in chromosome LcChr7 as are their orthologs in *Medicago* located in MtChr8 (*MtMLO13* and *MtMLO14*; *Medtr8g010500* and *Medtr8g010530*) (Fig 3, S5 Table). The two lentil genes plus the two of *Medicago* and the chickpea *CaMLO7* together form a subgroup within Clade II of the *MLO* sequences (Fig 5), suggesting that the duplication event predates the divergence of the tribes Vicieae, Cicereae and Trifolieae.

Phylogenetic analyses agree in classifying the *MLO* genes of legume species and other higher plant species into seven monophyletic clades [2, 20]. The *LcMLO* genes are distributed among these clades with the shortest genetic distances to pea, barrel medic and chickpea *MLOs*. Two or more sequences were included in the largest clades, I, II, III and V. Rispail and Rubiales [20] described that clade IV, initially thought to be restricted to monocots [2], contains one *MLO* per legume species. This is reinforced by the lentil results since only *LcMLO8* was included in this clade. Likewise, Rispail and Rubiales [20] pointed out that clade VI, characterized by the presence of *AtMLO3*, contains only genes from a small number of legume species, all of them from Phaseoloid legumes (soybean, common bean, mung bean and pingeon pea); again lentil results support this postulate since no lentil sequences were included in this clade.

In some non-legume species, *MLO* genes included in clade V have evidenced epistatic interactions in relation to the response to powdery mildew infection. Lentil *LcMLO1* and *LcMLO3* are included in this clade V and they share the highest similarities with *MtMLO1* and *AtMLO6* (S5 Table). In *Arabidopsis*, the *Atmlo2* single mutant displays a partial powdery mildew resistance, whereas the *Atmlo2/Atmlo6/Atmlo12* triple mutant is fully resistant [4]. In tomato (*Solanum lycopersicum*) the simultaneous silencing of *SlMLO1* and two of its closely related homologs, *SlMLO5* and *SlMLO8*, confers a higher resistance level than that associated with the *ol-2* mutation of *SlMLO1* [24]. Like *LcMLO1* and *LcMLO3*, the three epistatic genes of these two species are grouped in clade V [20, 24, 54]. In other species, however, full resistance is achieved with a single *MLO* mutant. In pea for instance, gene *er1* (*PsMLO1*) provides a complete or incomplete resistance depending on environmental conditions, and two additional genes, *er2* and *Er3*, are also involved in the powdery mildew resistance [31]. Thus, *LcMLO1* and *LcMLO3* are likely candidates to be involved in powdery mildew resistance in lentil, although whether the single loss-of-function mutants confer a complete or an incomplete resistance has to be checked.

Further sequences of these two likely candidates, *LcMLO1* and *LcMLO3*, were PCR amplified from other cultivated lentil materials and from the wild species of the genus *Lens*. Within each gene the sequences were very similar within and between species. Differences between sequences were mainly restricted to SNPs and small indels in introns (Fig 6). The whole genomic sequence of *LcMLO1* of CDC Redberry clearly differs from the sequences of the other cultivated *L*. *culinaris* tested as well as the wild *L*. *orientalis* sequences (S3 Fig) although the deduced amino acid sequences were identical (S1 Fig), since differences were limited to the introns. For MLO3 two amino acid substitutions between *L*. *culinaris* and *L*. *orientalis* were observed (S2 Fig). Amino acid substitutions and indels in the carboxyl intracellular domain were observed with respect to *L*. *odemensis*, *L*. *ervoides* and *L*. *lamottei* (S2 Fig).

Panstruga [55] described two conserved motifs in the C-terminus of 13 monocot and dicot MLO orthologs, down-stream of the calmodulin binding domain. The sequence of motif 1 in dicot species is TPTHG(S/M)SP(V/I)HLL(H/P) while the shorter motif 2 is (D/E)FSF. The candidate sequences in *Lens* species showed similar motifs: TPTHTMSPHLLH and DFSF in LcMLO1, and TPARSMSPAHVLPH and DEFSF in LcMLO3 (S1 and S2 Figs).

The function of *MLO* genes and the response to powdery mildew is mediated or influenced by other genes. The most characteristic examples are barley *Ror2* and *Ror1* (*Required for mlo-specified resistance*). *Ror1* and *Ror2* act by partially impairing *mlo* resistance, while the presence of *ror1* and *ror2* single mutants result in a super-susceptibility to *Blumeria graminis* f.sp. *hordei* when present in a susceptible *Mlo* background [13]. Likewise, in *Arabidopsis* the extracellular immune responses to ascomycete and oomycete pathogens are dependent on vesicle-associated secretion mediated by the SNARE proteins PEN1 syntaxin, SNAP33 and endomembrane-resident VAMP721/722 [16]. Thus, the identification of the homologous genes in lentil is interesting in relation to the response to powdery mildew and other fungi. For ROR2, all legume species analyzed showed a putative homologous gene (S4A Fig), except *M*. *truncatula* and *C*. *cajan* which showed a second sequence that grouped into a different cluster. Whether these sequences are homologous to the so far not well-characterized barley *Ror1* will need further analysis. *L*. *culinaris* and *L*. *odemensis* putative ROR2 (Syntaxin 122) shared sequence similarities and predicted functional characteristics with the barley ROR2: belonging to the SNARE superfamily with a Syntaxin N-terminal domain and a SNARE C-terminal domain which includes a transmembrane domain (from approximately 284 to 303) and function in the vesicle-mediated transport with a SNAP receptor activity as likely membrane components of the Golgi apparatus. Thus, the lentil *ROR2-like* gene is a possible candidate to be involved in the powdery mildew response.

For VAMP, the low supporting values of some tree nodes prevented us from obtaining definitive conclusions, but the results would support that VAMP721 and 722 are the result of a species-specific gene duplication in *Arabidopsis*, and that most of the analyzed legume species have genes coding for similar proteins to VAMP721 and 722 (S4B Fig). These *Lens* VAMP721/722 proteins shared predicted characteristics with the *Arabidopsis* proteins; i.e. the presence of an N-terminal login domain and a C-terminal synaptobrevin domain which overlap with a transmembrane domain at the C-end (from residue 196 to218 in *L*. *odemensis*, and 198 to 220 in cv. Alpo). They are considered as membrane components, acting in the vesicle-mediated transport. The location of a third sequence in *Lens* species, present as outgroup, suggests that these *Lens* sequences belong to different VAMP groups. Two putative homologs to *Arabidopsis* SNAP33 were found in the *Lens* species analyzed, while only one sequence per species was identified in all of the other legume species. All *Lens* VAMP-like proteins shared the functional characteristics of the *Arabidopsis* VAMP 721 and 722.

Sequences SNAP33-2 from *Lens* (Galegoid species) were clustered with a sequence of *C*. *cajan* (a Phaseoloid species) with a high node supporting value of 98, while sequences SNAP33-1 were clustered with sequences from all other legume species analyzed (Galegoid, Phaseoloid, Genistoid and Dalbergioid clades) also with high node supporting values (S4C Fig). These results raise the question whether the likely gene duplication of *SNAP33* is limited to the genus *Lens* (or perhaps to all of the Vicieae genera) and the sequence similarity with *C*. *cajan* is the consequence of convergent evolution, or else if the duplication is basal to the Papilonoid, where all of the four clades are included, and a gene has been lost in most of the species. Again, further research is needed to tackle these questions. In any case, this set of lentil *MLO*-related sequences are likely to be involved in the response to powdery mildew, and perhaps also to other fungi, thus being useful for breeding purposes.

## Conclusions

To sum up, in the lentil genome up to 15 functional genes and some pseudogenes of the *MLO* family have been identified. The sequences of the deduced proteins agree with the characteristics described for MLO proteins in other plant species such as the presence of transmembrane and calmodulin binding conserved domains. Phylogenetic analyses have indicated that *LcMLO1* and *LcMLO3* are the closest related to other genes involved in the response to powdery mildew present in other legume species and in other dicot plant species, in particular *LcMLO1* that shares some conserved motifs with these genes. Therefore these two genes are likely candidates to be involved in the response to this pathogen. We have designed useful primers to search in lentil germplasm collections for *LcMLO1* and *LcMLO3* deletion mutants as loss-of-function candidates likely conferring resistance to powdery mildew, although further investigation will be needed to confirm it. Data provided can be useful to lentil breeding programs as well as those in closely related species such as pea, grass pea or faba bean, providing more data to increase the knowledge of this interesting gene family.

## Supporting information

S1 Fig MLO1 sequence alignments of the Galegoid species. Alpo, Lupa and Redberry are cultivars of *L*. *culinaris* subsp. *culinaris*; Ps, *Pisum sativum*; Mt, *Medicago truncatula*; Ca, *Cicer arietinum*; the remaining names indicate the *Lens* species, or the subspecies *orientalis* of *L*. *culinaris*. Black lines indicate the transmembrane domains as defined by the CCTOP server (reliability = 88.81). Red line indicates the calmodulin binding domain as in Appiano et al. [1]. Green lines denote the two conserved motifs (1 and 2) of the C-terminal regions described by Panstruga [55].
(PDF)

S2 Fig MLO3 sequence alignments of the Galegoid species. Alpo, Lupa and Redberry are cultivars of *L*. *culinaris* subsp. *culinaris*; Mt, *Medicago truncatula*; Ca, *Cicer arietinum*; the remaining names indicate the *Lens* species, or the subspecies orientalis of *L*. *culinaris*. Black, red and green lines indicate the same domains and motifs as in the S1 Fig.
(PDF)

S3 Fig Neighbor-Joining phylogenetic trees of the lentil *LcMLO1* and *LcMLO3* genomic sequences. Whole nucleotide sequences (i.e, introns plus exons) were used to build trees using the Tamura two-parameter distance. Horizontal bar at bottom denotes the scale.
(PDF)

S4 Fig Neighbor-Joining phylogenetic trees of the proteins potentially related to MLO function. Amino acid sequences and the Jones-Taylor-Thornton distance with Gamma distribution were used to build trees. A) Proteins similar to ROR2 of *Hordeum vulgare*. B) Proteins similar to VAMP 721 and 722 of *Arabidopsis thaliana*. C) Proteins similar to SNAP33 of *A*. *thalina*. Horizontal bars denote the scale.
(PDF)

S5 Fig *Lens* Syntaxin 122 alignments with homologous sequences. Sequences of legume species, including *L*. *culinaris* and *L*. *odemensis*, compared to *A*. *thaliana* and *H*. *vulgare* sequences. Bottom horizontal lines indicate the predicted positions within of the *Lens* sequences of the Syntaxin domain (black), SNARE domain (red), and transmembrane domain (green). Intensity of the background blue color denotes similarity.
(PDF)

S1 Table Online prediction servers and tools used for the analysis of lentil *MLO* gene family. (PDF)

S2 Table Analyzed accessions of cultivated and wild relatives of lentil. (PDF)

S3 Table Sets of primers used for amplification of *MLO1* and *MLO3* genes. (PDF)

S4 Table Characteristics of full length lentil *MLO* genes. (DOCX)

S5 Table Protein characteristics and identity score of lentil MLOs with its closest homologues in *M*. *truncatula* and *A*. *thaliana*. (PDF)

S6 Table Conserved amino acids in MLO sequences according Elliot et al., 2005 [45]. (PDF)
