# Supplementary material for: A genome-wide identification and comparative analysis of the lentil MLO genes
Source: PLoS One. 2018 Mar 23;13(3):e0194945. doi: 10.1371/journal.pone.0194945 (PMC5865747; doi:10.1371/journal.pone.0194945)
Supplement: S1 Fig — Alpo, Lupa and Redberry are cultivars of L. culinaris subsp. culinaris; Ps, Pisum sativum; Mt, Medicago truncatula; Ca, Cicer arietinum; the remaining names indicate the Lens species, or the subspecies orientalis of L. culinaris. Black lines indicate the transmembrane domains as defined by the CCTOP server (reliability = 88.81). Red line indicates the calmodulin binding domain as in Appiano et al. [1]. Green lines denote the two conserved motifs (1 and 2) of the C-terminal regions described by Panstruga [55]. (PDF) [file pone.0194945.s001.pdf]

S1 Fig

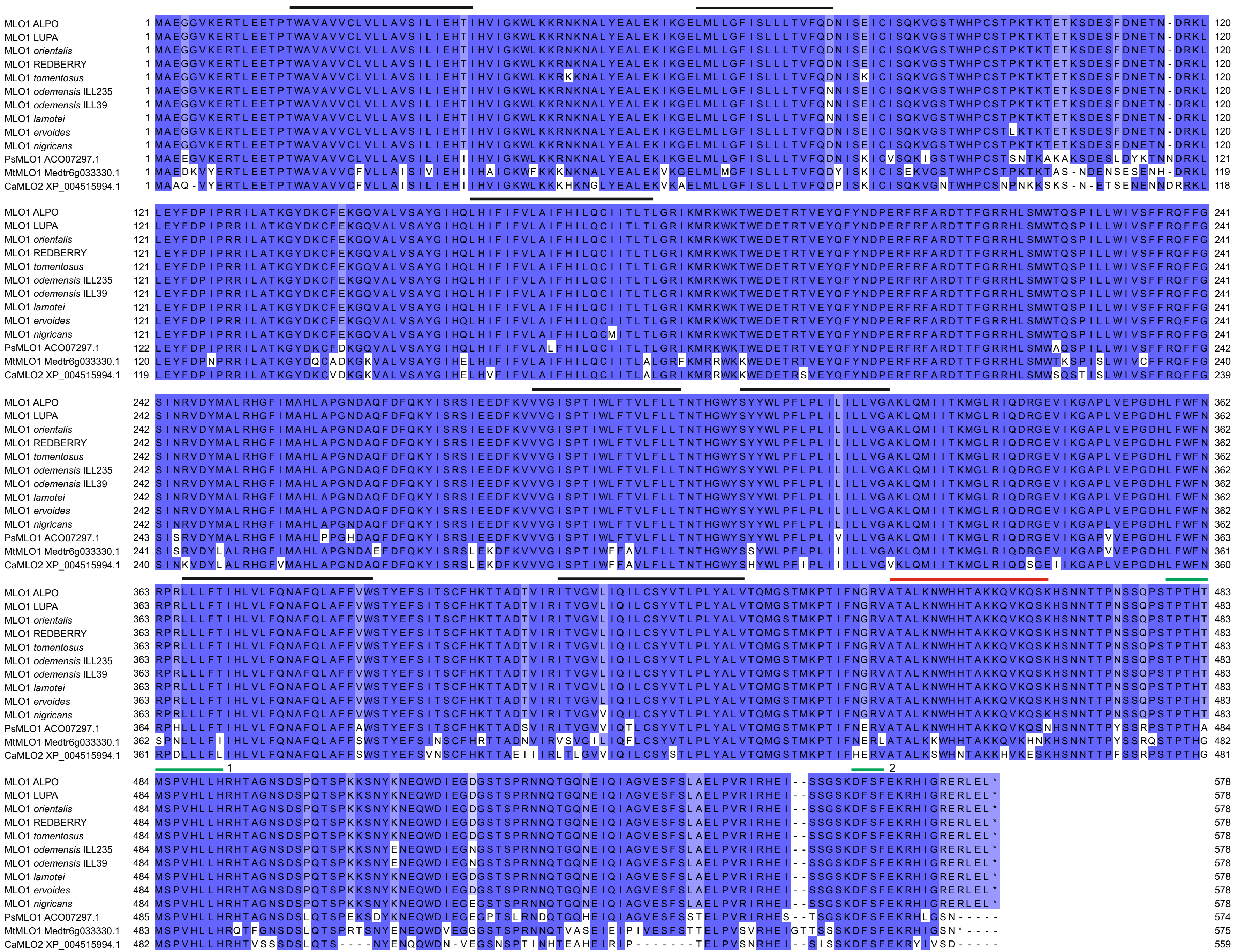

MLO1 ALPO
MLO1 LUPA
MLO1 orientalis
MLO1 REDBERRY
MLO1 tomentosus
MLO1 odemensis ILL235
MLO1 odemensis ILL39
MLO1 lamotei
MLO1 ervoides
MLO1 nigricans
PsMLO1 ACO07297.1
MtMLO1 Medtr6g033330.1
CaMLO2 XP_004515994.1
1
2
