# Supplementary material for: A genome-wide identification and comparative analysis of the lentil MLO genes
Source: PLoS One. 2018 Mar 23;13(3):e0194945. doi: 10.1371/journal.pone.0194945 (PMC5865747; doi:10.1371/journal.pone.0194945)
Supplement: S2 Fig — Alpo, Lupa and Redberry are cultivars of L. culinaris subsp. culinaris; Mt, Medicago truncatula; Ca, Cicer arietinum; the remaining names indicate the Lens species, or the subspecies orientalis of L. culinaris. Black, red and green lines indicate the same domains and motifs as in the S1 Fig. (PDF) [file pone.0194945.s002.pdf]

S2 Fig

```
MLO3 ALPO                 1 MAGEGGGRSLTETPTWAVAVVCFVLLFISIIIEHIFHFIGNWLKKRHKSALYESLEKIKSELMLLGFISLLLTVGQGLISKICISEKVAATWHPCSNNAAKGEDS----EDESGGR-RRLLAD--------PR 120
MLO3 LUPA                 1 MAGEGGGRSLTETPTWAVAVVCFVLLFISIIIEHIFHFIGNWLKKRHKSALYESLEKIKSELMLLGFISLLLTVGQGLISKICISEKVAATWHPCSNNAAKGEDS----EDESGGR-RRLLAD--------PR 120
MLO3 REDBERRY             1 MAGEGGGRSLTETPTWAVAVVCFVLLFISIIIEHIFHFIGNWLKKRHKSALYESLEKIKSELMLLGFISLLLTVGQGLISKICISEKVAATWHPCSNNAAKGEDS----EDESGGR-RRLLAD--------PR 120
MLO3 orientalis          1 MAGEGGGRSLTETPTWAVAVVCFVLLFISIIIEHIFHFIGNWLKKRQKSALYESLEKIKSELMLLGFISLLLTVGQGLISKICISEKVAATWHPCSNNAAKGEDS----EDESGGR-RRLLAD--------PR 120
MLO3 lamotei             1 MAGEGGGRSLTETPTWAVAVVCFVILFISIIIEHIFHFIGNWLKKRHKSALYESLEKIKSELMLLGFISLLLTVGQGLISKICISEKVAATWHPCSNNAAKGEDS----EDESGGR-RRLLAD--------PR 120
MLO3 odemensis ILL235   1 MAGEGGGRSLTETPTWAVAVVCFVILFISIIIEHIFHFIGNWLKKRHKSALYESLEKIKSELMLLGFISLLLTVGQGLISKICISEKVAATWHPCSNNAAKGEDS----EDESGGR-RRLLAD--------SR 120
MLO3 odemensis ILL39    1 MAGEGGGRSLTETPTWAVAVVCFVILFISIIIEHIFHFIGNWLKKRHKSALYESLEKIKSELMLLGFISLLLTVGQGLISKICISEKVAATWHPCSNNAAKGEDS----EDESGAR-RRLLAD--------PR 120
MLO3 tomentosus          1 MAGEGGGRSLTETPTWAVAVVCFVLLFISIIIEHIFHFIGNWLKKRHKSALYESLEKIKSELMLLGFISLLLTVGQGLISKICISEKVAATWHPCSNNAAKGEDS----EGESGGR-RRLLAD--------PR 120
MLO3 ervoides            1 MAGEGGGRSLTETPTWAVAVVCFVLLFISIIIEHIFHFIGNWLKKRHKNALYESLEKIKSELMLLGFISLLLTVGQGLISKICISEKVAATWHPCSNNAAKGEDS----EDESGGR-RRLLAD--------PR 120
MLO3 nigricans           1 MAGEGGGRSLTETPTWAVAVVCFVLLFISIIIEHIFHFIGNWLKKRHKSALHESLEKIKSELMLLGFISLLLTVGQGLISKICISEKVAATWHPCSNNAAKGEDS----EDESGGHHRRLLAD--------PR 121
CaMLO6 XP_004487134.1     1 MAG---GRSLTETPTWAVAVVCFVILSISIIIEHIFHIIGKWLKNKHKSALYESLEKIKSELMLLGFISLLLTIGEGLISKICISEKVAATWHPCTNNAERNSDDHQPIEHETGG--RRLLAALLASQNKNSR 128
MtMLO3 Medtr2g093750.1    1 MAGGSVGRSLTETPTWAVAVVCFVILSISIFIEHIFHIIEKWLKKKHKSALYESLEKIKSELMLLGFISLLLTVGEGLISRICISEKVAATWHPCSNNANIESDDEELIDHETGGS-RRLLAALLASQGDNHH 132

MLO3 ALPO               121 RILAGGGFDKCAENGKVAFVSADGIHQLHIFIFVLAVFHVLYCILTLALGRAKMRRWKRWEEETKTLEYQFSHDPDRFRFANETSFGRRHLSFWTKNPILIWIVCFFRQFVRSVPEVDYLTLRHGFMMAHLAP 253
MLO3 LUPA               121 RILAGGGFDKCAENGKVAFVSADGIHQLHIFIFVLAVFHVLYCILTLALGRAKMRRWKRWEEETKTLEYQFSHDPDRFRFANETSFGRRHLSFWTKNPILIWIVCFFRQFVRSVPEVDYLTLRHGFMMAHLAP 253
MLO3 REDBERRY           121 RILAGGGFDKCAENGKVAFVSADGIHQLHIFIFVLAVFHVLYCILTLALGRAKMRRWKRWEEETKTLEYQFSHDPDRFRFANETSFGRRHLSFWTKNPILIWIVCFFRQFVRSVPEVDYLTLRHGFMMAHLAP 253
MLO3 orientalis        121 RILAGGGFDKCAENGKVAFVSADGIHQLHIFIFVLAVFHVLYCILTLALGRAKMRRWKRWEEETKTLEYQFSHDPDRFRFANETSFGRRHLSFWTKNPILIWIVCFFRQFVRSVPEVDYLTLRHGFMMAHLAP 253
MLO3 lamotei           121 RILGGGGFDKCAENGKVAFVSADGIHQLHIFIFVLAVFHVLYCILTLALGRAKMRRWKRWEEETKTLEYQFSHDPDRFRFANETSFGRRHLSFWTKNPILIWIVCFFRQFVRSVPEVDYLTLRHGFMMAHLAP 253
MLO3 odemensis ILL235 121 RILAGGGFDKCAENGKVAFVSADGIHQLHIFIFVLAVFHVLYCILTLALGRAKMRRWKRWEEETKTLEYQFSHDPDRFRFANETSFGRRHLSFWTKNPILIWIVCFFRQFVRSVPEVDYLTLRHGFMMAYLAP 253
MLO3 odemensis ILL39  121 RILAGGGFDKCAENGKVAFVSADGIHQLHIFIFVLAVFHVLYCILTLALGRAKMRRWKRWEEETKTLEYQFSHDPDRFRFANETSFGRRHLSFWTKNPILIWIVCFFRQFVRSVPEVDYLTLRHGFMMAYLAP 253
MLO3 tomentosus        121 RILAGGGFDKCAENGKVAFVSADGIHQLHIFIFVLAVFHVLYCILTLALGRAKMRRWKRWEEETKTLEYQFSHDPDRFRFANETSFGRRHLSFWTKNPILIWIVCFFRQFVRSVPEVDYLTLRHGFMMAHLEP 253
MLO3 ervoides          121 RILAGGGFDKCAENGKVAFVSADGIHQLHIFIFVLAVFHVLYCILTLALGRAKMRRWKRWEEETKTLEYQFSHDPDRFRFANETSFGRRHLSFWTKNPILIWIVCFFRQFVRSVPEVDYLTLRHGFMMAHLAP 253
MLO3 nigricans         122 RILAGGGFDKCAENGKVAFVSADGIHQLHIFIFVLAVFHVLYCILTLALGRAKMRRWKRWEEETKTLEYQFSHDPDRFRFANETSFGRRHLSFWTKNPILIWIVCFFRQFVRSVPEVDYLTLRHGFMMAHLAP 254
CaMLO6 XP_004487134.1   129 RILAAGGIDKCAAQGKVAFVSSEGIHQLHIFIFVLAVFHVLYCILTLALGRAKMRRWKRWEEETKTLEYQFSHDPERFRFASETSFGRRHLSFWTKNPVLIWIVCFFRQFVRSVPEVDYLTLRHGFMMAHLAP 261
MtMLO3 Medtr2g093750.1  133 RILAGGGGDKCAEEGKVAFVSAGAIHQLHIFIFVLAVFHILYCILTLALGRAKMRRWKRWEEATKTPEYQFSHDPERFRFANETSFGRRHLSFWTKNPVLIWIVCFFRQFVRSVPEVDYLTLRHGFMMAHLAP 265

MLO3 ALPO               254 SSHQKFDFRQYIKRCLEEDFKVVVEISPPLWFIAVFFLLFHTHGWYSYLWLPFVPLIIVLLVGTKLQVIITQMGLRIQKQGMVVKGEPVVQPGDDLFWFNKPRLILYLINFVLFQNAFQLAFFSWTAFQFGLK 386
MLO3 LUPA               254 SSHQKFDFRQYIKRCLEEDFKVVVEISPPLWFIAVFFLLFHTHGWYSYLWLPFVPLIIVLLVGTKLQVIITQMGLRIQKQGMVVKGEPVVQPGDDLFWFNKPRLILYLINFVLFQNAFQLAFFSWTAFQFGLK 386
MLO3 REDBERRY           254 SSHQKFDFRQYIKRCLEEDFKVVVEISPPLWFIAVFFLLFHTHGWYSYLWLPFVPLIIVLLVGTKLQVIITQMGLRIQKQGMVVKGEPVVQPGDDLFWFNKPRLILYLINFVLFQNAFQLAFFSWTAFQFGLK 386
MLO3 orientalis        254 SSHQKFDFRQYIKRCLEEDFKVVVEISPPLWFIAVFFLLFHTHGWYSYLWLPFVPLIIVLLVGTKLQVIITQMGLRIQKQGTVVKGEPVVQPGDDLFWFNKPRLILYLINFVLFQNAFQLAFFSWTAFQFGLK 386
MLO3 lamotei           254 SSHQKFDFRQYIKRCLEEDFKVVVEISPPLWFIAVFFLLFHTHGWYSYLWLPFVPLIIVLLVGTKLQVIITQMGLRIQKQGMVVKGEPVVQPGDDLFWFNKPRLILYLINFVLFQNAFQLAFFSWTAFQFGLK 386
MLO3 odemensis ILL235 254 SSHQKFDFRQYIKRCLEEDFKVVVEISPPLWFIAVFFLLFHTHGWYSYLWLPFVPLIIVLLVGTKLQVIITQMGLRIQKQGMVVKGEPVVQPGDDLFWFNKPRLILYLINFVLFQNAFQLAFFSWTAFQFGLK 386
MLO3 odemensis ILL39  254 LSHQKFDFRQYIKRCLEEDFKVVVEISPPLWFIAVFFLLFHTHGWYSYLWLPFVPLIIVLLVGTKLQVIITQMGLRIQKQGMVVKGEPVVQPGDDLFWFNKPRLILYLINFVLFQNAFQLAFFSWTAFQFGLK 386
MLO3 tomentosus        254 LSHQKFDFRQYIKRCLEEDFKVVGEISPPLWFIAVFFLLFHTHGWYSYLWLPFVPLIIVLLVGTKLQVIITQMGLRIQKQGMVVKGEPVVQPGDDLFWFNKPRLILYLINFVLFQNAFQLAFFSWTAFQFGLK 386
MLO3 ervoides          254 SSHKKFDFRQYIKRCLEEDFKVVVEISPPLWFIAVFFLLFHTHGWYSYLWLPFVPLIIVLLVGTKLQVIITQMGLRIQKQGMVVKGEPVVQPGDDLFWFNKPRLILYLINFVLFQNAFQLAFFSWTAFQFGLK 386
MLO3 nigricans         255 SSHQKFDFRQYIKRCLEEDFKVVVEISPPLWFIAVFFLLFYTHGWNSYLWLPFVPLIIVLLVGTKLQVIITQMGLRIQKQGMVVKGEPVVQPGDDLFWFNKPRLILYLINFVLFQNAFQLAFFSWTAFQFGLK 387
CaMLO6 XP_004487134.1   262 QSHQKFDFRHYIKRCLEEDFKVVVEISPPIWFITVLFLLFHTHGWHSYLWLPFVPLIIVLLVGTKLQVIITQMGLRIQKQGMVVKGEPVVQPGDDLFWFSKPRLILHLINFVLFQNAFQLAFFSWTALQFGLT 394
MtMLO3 Medtr2g093750.1  266 QSHLKFDFRQYIKRCLEEDFKVVVGISPPIWFITVFFLLFHTHGWHSYLWLPFLPLIIVLLVGTKLQVIITQMGLRIQKQGMVVKGEPVVQPRDDLFWFNKPRLILFLINFVLFQNAFQLAFFSWTALQFGVT 398

[1]

MLO3 ALPO               387 SCYNSRSEDVVIRISMGIIVQILCSYVTLPLYALVTQMGSTMKPTIFNERVATALKHWHHNAKKHIKQNRGPGFQSPMTSRSITPARSMSPAHVLPHCRSEIDLS--------------PYESNSPSPSNSHH 505
MLO3 LUPA               387 SCYNSRSEDVVIRISMGIIVQILCSYVTLPLYALVTQMGSTMKPTIFNERVATALKHWHHNAKKHIKQNRGPGFQSPMTSRSITPARSMSPAHVLPHCRSEIDLS--------------PYESNSPSPSNSHH 505
MLO3 REDBERRY           387 SCYNSRSEDVVIRISMGIIVQILCSYVTLPLYALVTQMGSTMKPTIFNERVATALKHWHHNAKKHIKQNRGPGFQSPMTSRSITPARSMSPAHVLPHCRSEIDLS--------------PYESNSPSPSNSHH 505
MLO3 orientalis        387 SCYNSRSEDVVIRISMGIIVQILCSYVTLPLYALVTQMGSTMKPTIFNERVATALKHWHHNAKKHIKQNRGPGFQSPMTSRSITPARSMSPAHVLPHCRSEIDLS--------------PYESNSPSPSNSHH 505
MLO3 lamotei           387 SCYNSRSEDVVIRISMGIIVQILCSYVTLPLYALVTQMGSTMKPTIFNERVATALKHWHHNAKKHIKQNRGPGFQSPMTSRPITPARSMSPAHVLPHCRSEIDLS--------------PYESNSPSPSNSHH 505
MLO3 odemensis ILL235 387 SCYNSRSEDVVIRISMGIIVQILCSYVTLPLYALVTQMGSTMKPTIFNERVATALKHWHHNAKKHIKQNRGPGFQSPMTSRSITPARSMSPAHVLPHCRSEIDLS--------------PYESNSPSPSNSHH 505
MLO3 odemensis ILL39  387 SCYNSRSEDVVIRISMGIIVQILCSYVTLPLYALVTQMGSTMKPTIFNERVATALKHWHHNAKKHIKQNRGPGFQSPMTSRSITPARSMSPAHVLPHCRSEIDLS--------------PYESNSPSPSNSHH 505
MLO3 tomentosus        387 SCYNSRSEDVVIRISMGIIVQILCSYVTLPLYALVTQMGSTMKPTIFNERVATALKHWHHNAKKHIKQNRGPGFQSPMTSRPITPARSMSPAHVLPHCRSEIDLS--------------PYESNSPSPSNSHH 505
MLO3 ervoides          387 SCYNSRSEDVVIRISMGIIVQILCSYVTLPLYALVTQMGSTMKPTIFNERVATALKHWHHNAKKHIKQNRGPGFQSPMTSRPITPARSMSPAHVLPHCRSEIDLS--------------PYESNSPSPSNSHH 505
MLO3 nigricans         388 SCYNSRSEDVVIRISMGIIVQILCSYVTLPLYALVTQMGSTMKPTIFNERVATALKHWHHNAKKHIKQNRGPGFQSPMTSRPITPARSMSPAHVLRHCRSEIDLS--------------PYESNSPSPSNSHH 506
CaMLO6 XP_004487134.1   395 SCFNSRTEDVVIRISMGVFVQILCSYVTLPLYALVTQMGSTMKPAIFNERVATALRNWHNTAKKHIKQNRGSGFQSPMSTRSITPAHSMSPAHILRPYHSETDTHPTTSPTRLNFETYNPYEAYSPSPSN--- 524
MtMLO3 Medtr2g093750.1  399 SCYNSRKDGVVIRICMGVFVQILCSYVTLPLYALVTQMGSTMKPTIFNERVATALRNWHHTARKNIKHNRGSGSQTPFSSRSITPARSMSPAQILRHYRNQMDT-----PTRLNFETSHHYESYSPSPSNSHH 526

[2]

MLO3 ALPO               506 HKVET---SSGSIILHEMEMGHLARDKEQEIVGPNCVSVGLGRPRFVVDVQHSDEFSFSKMATNQLE* 570
MLO3 LUPA               506 HKVET---SSGSIILHEMEMGHLARDKEQEIVGPNCVSVGLGRPRFVVDVQHSDEFSFSKMATNQLE* 570
MLO3 REDBERRY           506 HKVET---SSGSIILHEMEMGHLARDKEQEIVGPNCVSVGLGRPRFVVDVQHSDEFSFSKMATNQLE* 570
MLO3 orientalis        506 HKVET---SSGSIILHEMEMGHLARDKEQEIVGPNCVSVGLGRPRFVVDVQHSDEFSFSKMATNQLE* 570
MLO3 lamotei           506 HKVETGAASSGSIILHEMEMGHLARDKEQEIVEPNCVSVGLGRPQFVVDVQHSDEFSFSKMATNQLE* 573
MLO3 odemensis ILL235 506 HKVETGAASSGSIILHEMEMGYLARDKEQEIVGPNCVSVGLGRPQFVVDVQHSDEFSFSKMATNQLE* 573
MLO3 odemensis ILL39  506 HKVET---SSGLIILHEMEMGHLARDKEQEIVEPNCVSVGLGRPQFVVDVQHSDEFSFSKMATNQLE* 570
MLO3 tomentosus        506 HKVET---SSGSIILHEMEMGHLARDKEQEIVGPNCVSVGLGRPRFVVDVQHSDEFSFSKMATNQLE* 570
MLO3 ervoides          506 HKVETGVASSGSIILHEMEMGHLARDKEQEIGEPNCVSVGLGRPQFVVDVQHSDEFSFSKMATNQLE* 573
MLO3 nigricans         507 HKVET---SSGSIILHEMEMGHLARDKEQEIVEPNCVSVGLGRPQFVVDVQHSDEFSFSKMATNQLE* 571
CaMLO6 XP_004487134.1   525 -KVEARATSSSSIYFHEMEMGHLAHDHEQETNKPNCVSVGSGLTQLEIDVQHSDEFSFSKMATNNLK- 590
MtMLO3 Medtr2g093750.1  527 HKVEINVASSSSTHLHEMEMGHLAHVEQQEVIKPNSISVGSGRPQFEIDIQQSDELSFSTMPTNQLE* 594
```
