# Supplementary figures and images for: A genome-wide identification and comparative analysis of the lentil MLO genes
Source: PLoS One. 2018 Mar 23;13(3):e0194945. doi: 10.1371/journal.pone.0194945 (PMC5865747; doi:10.1371/journal.pone.0194945)

S3 Fig

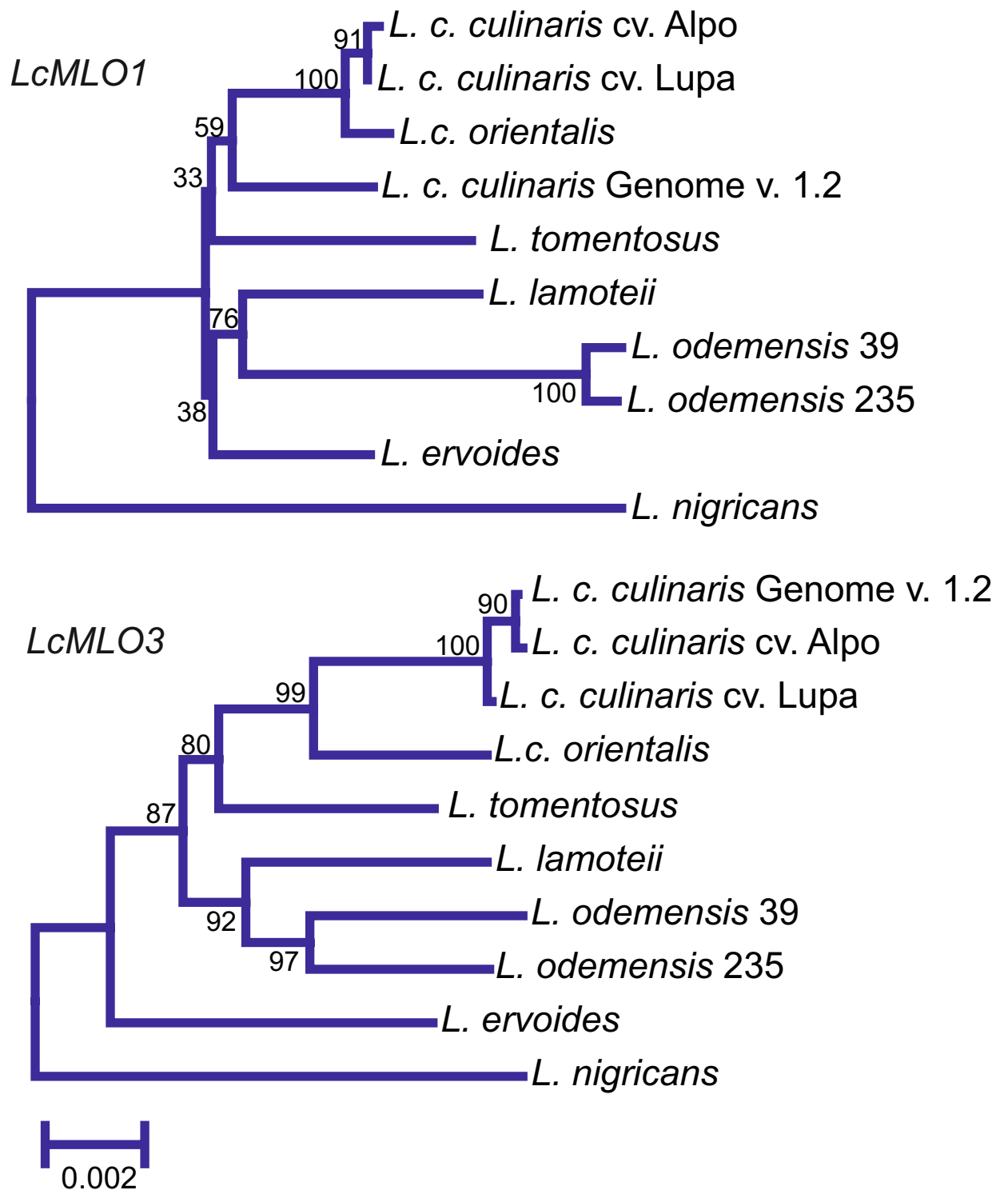

Supplement: S3 Fig — Whole nucleotide sequences (i.e, introns plus exons) were used to build trees using the Tamura two-parameter distance. Horizontal bar at bottom denotes the scale. (PDF) [file pone.0194945.s003.pdf]

# S4 Fig

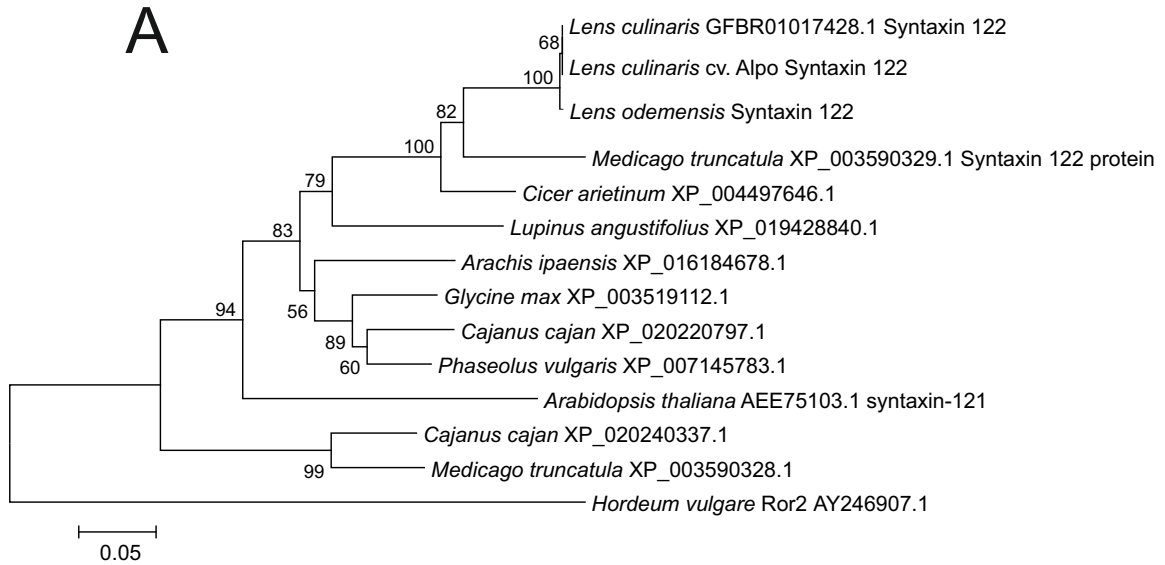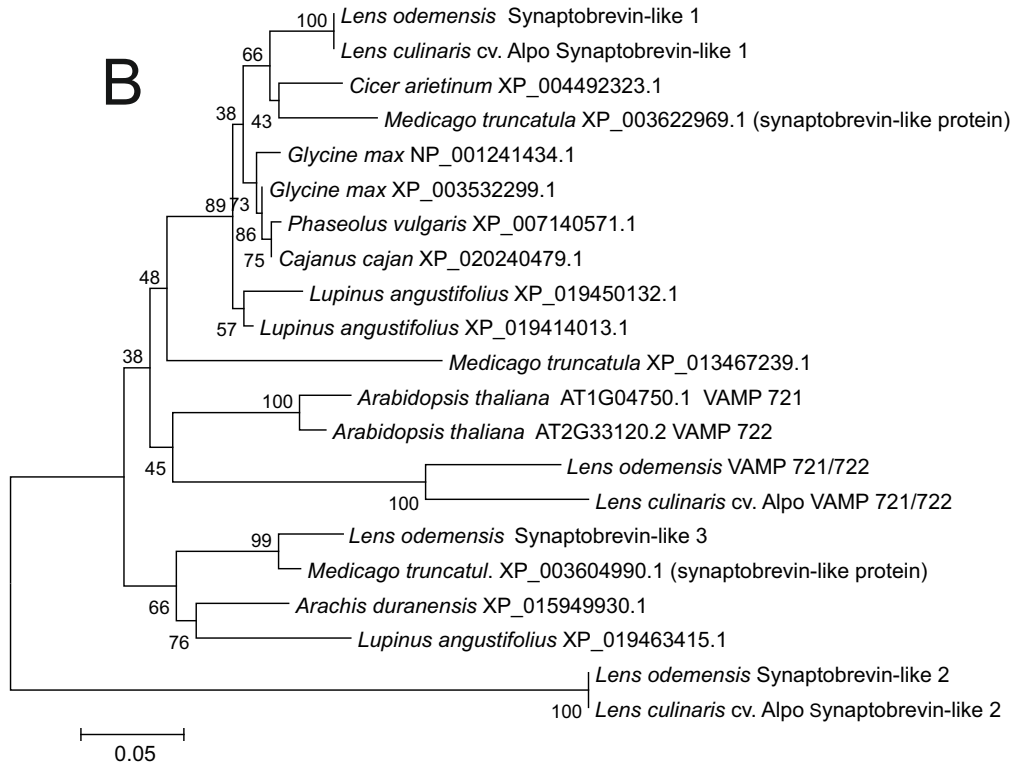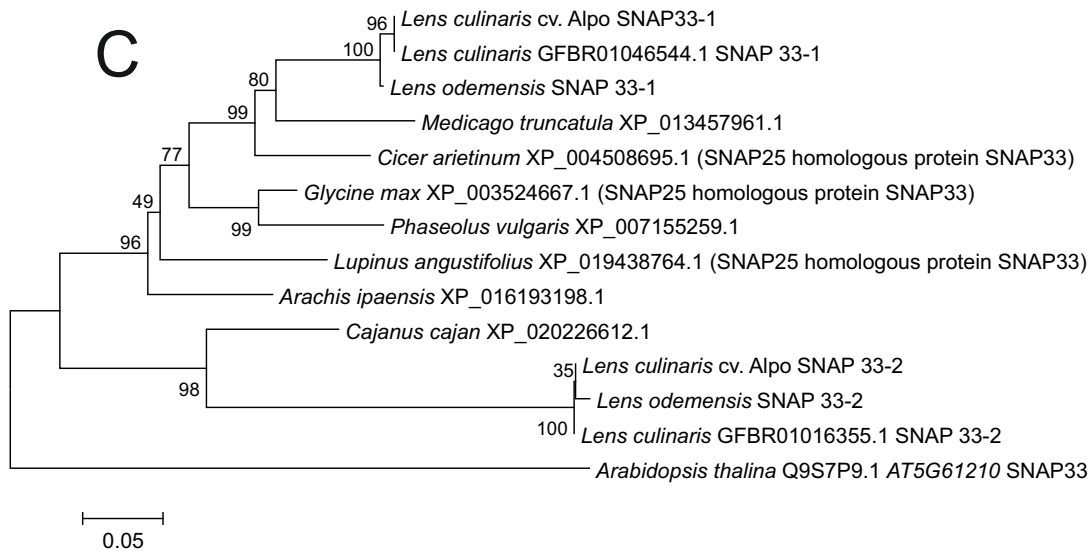

Supplement: S4 Fig — Amino acid sequences and the Jones-Taylor-Thornton distance with Gamma distribution were used to build trees. A) Proteins similar to ROR2 of Hordeum vulgare. B) Proteins similar to VAMP 721 and 722 of Arabidopsis thaliana. C) Proteins similar to SNAP33 of A. thalina. Horizontal bars denote the scale. (PDF) [file pone.0194945.s004.pdf]
