# Supplementary material for: A genome-wide identification and comparative analysis of the lentil MLO genes
Source: PLoS One. 2018 Mar 23;13(3):e0194945. doi: 10.1371/journal.pone.0194945 (PMC5865747; doi:10.1371/journal.pone.0194945)
Supplement: S5 Fig — Sequences of legume species, including L. culinaris and L. odemensis, compared to A. thaliana and H. vulgare sequences. Bottom horizontal lines indicate the predicted positions within of the Lens sequences of the Syntaxin domain (black), SNARE domain (red), and transmembrane domain (green). Intensity of the background blue color denotes similarity. (PDF) [file pone.0194945.s005.pdf]

S5 Fig

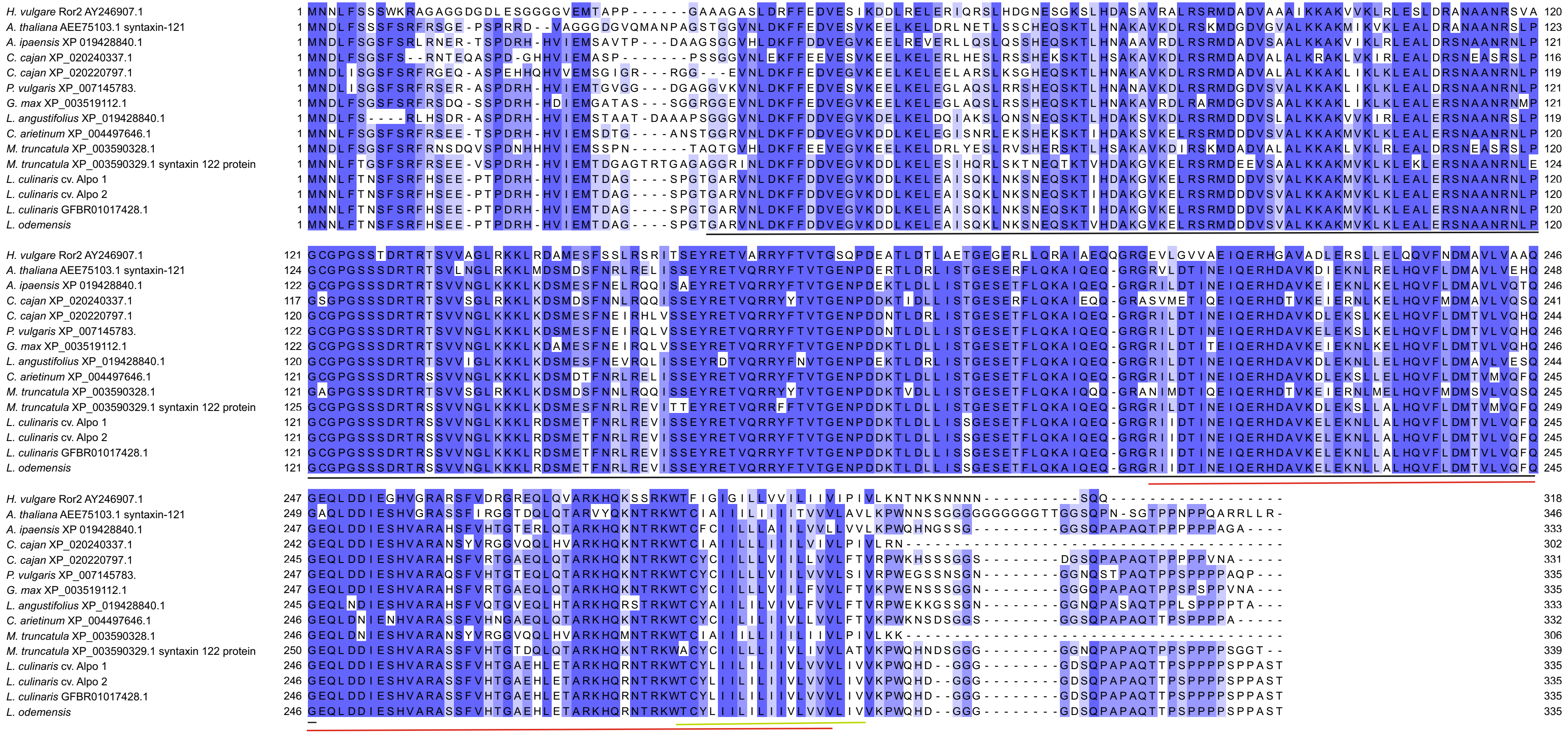

H. vulgare Ror2 AY246907.1 1 MNNLFSSSWKRAGAGGDGDLESGGGGVEMTAPP-----GAAAGASLDRFFEDVESIKDDLRELERIQRSLHDGNESGKSLHDASAVRALRSRMDADVAAAIKKAKVVKLRLESLDRANAANRSVA 120
A. thaliana AEE75103.1 syntaxin-121 1 MNDLFSSSFSRFRSGE-PSPRRD--VAGGGDGVQMANPAGSTGGVNLDKFFEDVESVKEELKELDRLNETLSSCHEQSKTLHNAKAVKDLRSKMDGDVGVALKKAKMIKVKLEALDRANAANRSLP 123
A. ipaensis XP 019428840.1 1 MNDLFSGSFSRLRNER-TSPDRH-HVIEMSAVTP---DAAGSGGVHLDKFFDDVEGVKEELREVERLLQSLQSSHEQSKTLHNAAVRDLRSRMDADVSAALKKAKVIKLRLEALDRSNAANRNLP 121
C. cajan XP_020240337.1 1 MNDLFSGSFS--RNTEQASPD-GHHVIEMASP-------PSSGGVNLEKFFEEVESVKEELKELERLHESLRSSHEKSKTLHSAKAVKDLRSRMDADVALALKRAKLVKIRLEALDRSNEASRSLP 116
C. cajan XP_020220797.1 1 MNDLISGSFSRFRGEQ-ASPEHHQHVVEMSGIGR---RGG---EVNLDKFFEDVEGVKEELKELEELARSLKSGHEQSKTLHNAKAVKDLRSRMDADVALALKKAKLIKLKLEALDRSNAANRNLP 119
P. vulgaris XP_007145783. 1 MNDLISGSFSRFRSER-ASPDRH-HVIEMTGVGG---DGAGGVKVNLDKFFEDVESVKEELKELEGLAQSLRRSHEQSKTLHNANAVKDLRSRMDGDVSVALKKAKLIKLKLEALDRSNAANRNLP 121
G. max XP_003519112.1 1 MNDLFSGSFSRFRSDQ-SSPDRH-HDIEMGATAS---SGGRGGEVNLDKFFEDVEGVKEELKELEGLAQSLRSSHEQSKTLHNAKAVRDLRARMDGDVSAALKKAKLIKLKLEALERSNAANRNMP 121
L. angustifolius XP_019428840.1 1 MNDLFS----RLHSDR-ASPDRH-HVIEMSTAAT-DAAAPSGGGVNLDKFFDDVEGVKDELKELDQIAKSLQNSNEQSKTLHDAKSVKDLRSRMDDDVSLALKKAKVVKIRLEALERSNAANRSLP 119
C. arietinum XP_004497646.1 1 MNNLFSGSFSRFRSEE-TSPDRH-HVIEMSDTG----ANSTGGRVNLDKFFDDVEGVKDDLKELEGISNRLEKSHEKSKTIHDAKSVKELRSRMDDDVSVALKKAKMVKLKLEALERSNAANRNLP 120
M. truncatula XP_003590328.1 1 MNDLFSGSFSRFRNSDQVSPDNHHHVIEMSSPN------TAQTGVHLDKFFEEVEGVKEELKELDRLYESLRVSHERSKTLHSAKAVKDIRSKMDADVALALKKAKLVKLKLEALDRSNEASRSLP 120
M. truncatula XP_003590329.1 syntaxin 122 protein 1 MNNLFTGSFSRFRSEE-VSPDRH-HVIEMTDGAGTRTGAGAGGRINLDKFFDDVEGVKDDLKELESIHQRLSKTNEQTKTVHDAKGVKELRSRMDEEVSAALKKAKMVKLKLEKLERSNAANRNLE 124
L. culinaris cv. Alpo 1 1 MNNLFTNSFSRFHSEE-PTPDRH-HVIEMTDAG----SPGTGARVNLDKFFDDVEGVKDDLKELEAISQKLNKSNEQSKTIHDAKGVKELRSRMDDDVSVALKKAKMVKLKLEALERSNAANRNLP 120
L. culinaris cv. Alpo 2 1 MNNLFTNSFSRFHSEE-PTPDRH-HVIEMTDAG----SPGTGARVNLDKFFDDVEGVKDDLKELEAISQKLNKSNEQSKTIHDAKGVKELRSRMDDDVSVALKKAKMVKLKLEALERSNAANRNLP 120
L. culinaris GFBR01017428.1 1 MNNLFTNSFSRFHSEE-PTPDRH-HVIEMTDAG----SPGTGARVNLDKFFDDVEGVKDDLKELEAISQKLNKSNEQSKTIHDAKGVKELRSRMDDDVSVALKKAKMVKLKLEALERSNAANRNLP 120
L. odemensis 1 MNNLFTNSFSRFHSEE-PTPDRH-HVIEMTDAG----SPGTGARVNLDKFFDDVEGVKDDLKELEAISQKLNKSNEQSKTVHDAKGVKELRSRMDDDVSVALKKAKMVKLKLEALERSNAANRNLP 120
H. vulgare Ror2 AY246907.1 121 GCGPGSSTDRTRTSVVAGLRKKLRDAMESFSSLRSRITSEYRETVARRYFTVTGSQPDEATLDTLAETGEGERLLQRAIAEQQGRGEVLGVVAEIQERHGAVADLERSLLELQQVFNDMAVLVAAQ 246
A. thaliana AEE75103.1 syntaxin-121 124 GCGPGSSSDRTRTSVLNGLRKKLMDSMDSFNRLRELISSEYRETVQRRYFTVTGENPDERTLDRLISTGESERFLQKAIQEQ-GRGRVLDTINEIQERHDAVKDIEKNLRELHQVFLDMAVLVEHQ 248
A. ipaensis XP 019428840.1 122 GCGPGSSSDRTRTSVVNGLKKKLKDSMESFNELRQQISAEYRETVQRRYFTVTGENPDEKTLDLLISTGESETFLQKAIQEQ-GRGRILDTINEIQERHDAVKEIEKNLKELHQVFLDMAVLVQTQ 246
C. cajan XP_020240337.1 117 GSGPGSSSDRTRTSVVSGLRKKLKDSMDSFNNLRQQISSEYRETVQRRYYTVTGENPDDKTIDLLISTGESERFLQKAIEQQ-GRASVMETIQEIQERHDTVKEIERNLKELHQVFMDMAVLVQSQ 241
C. cajan XP_020220797.1 120 GCGPGSSSDRTRTSVVNGLKKKLKDSMESFNEIRHLVSSEYRETVQRRYFTVTGENPDDNTLDRLISTGESETFLQKAIQEQ-GRGRILDTINEIQERHDAVKDLEKSLKELHQVFLDMTVLVQHQ 244
P. vulgaris XP_007145783. 122 GCGPGSSSDRTRTSVVNGLKKKLKDSMESFNEIRQLVSSEYRETVQRRYFTVTGENPDDNTLDLLISTGESETFLQKAIQEQ-GRGRILDTINEIQERHDAVKELEKSLKELHQVFLDMTVLVQHQ 246
G. max XP_003519112.1 122 GCGPGSSSDRTRTSVVNGLKKKLKDAMESFNEIRQLVSSEYRETVQRRYFTVTGENPDDKTLDLLISTGESETFLQKAIQEQ-GRGRILDTITEIQERHDAVKEIEKNLKELHQVFLDMTVLVQHQ 246
L. angustifolius XP_019428840.1 120 GCGPGSSSDRTRTSVVIGLKKKLKDSMESFNEVRQLISSEYRDTVQRRYFNVTGENPDEKTLDRLISTGESETFLQKAIQEQ-GRGRILDTINEIQERHDAVKDLEKNLLELHQVFLDMAVLVESQ 244
C. arietinum XP_004497646.1 121 GCGPGSSSDRTRSSVVNGLKKKLKDSMDTFNRLRELISSEYRETVQRRYFTVTGENPDDKTLDLLISTGESETFLQKAIQEQ-GRGRILDTINEIQERHDAVKDLEKSLLELHQVFLDMTVMVQFQ 245
M. truncatula XP_003590328.1 121 GAGPGSSSDRTRTSVVSGLRKKLKDSMDSFNNLRQQISSEYRETVQRRYYTVTGENPDDKTVDLLISTGESETFLQKAIQQQ-GRANIMDTIQEIQERHDTVKEIERNLMELHQVFMDMSVLVQSQ 245
M. truncatula XP_003590329.1 syntaxin 122 protein 125 GCGPGSSSDRTRSSVVNGLKKKLKDSMESFNRLREVITTEYRETVQRRFFTVTGENPDDKTLDLLISTGESETFLQKAIQEQ-GRGRILDTINEIQERHDAVKDLEKSLLALHQVFLDMTVMVQFQ 249
L. culinaris cv. Alpo 1 121 GCGPGSSSDRTRSSVVNGLKKKLRDSMETFNRLREVISSEYRETVQRRYFTVTGENPDDKTLDLLISSGESETFLQKAIQEQ-GRGRIIDTINEIQERHDAVKELEKNLLALHQVFLDMTVLVQFQ 245
L. culinaris cv. Alpo 2 121 GCGPGSSSDRTRSSVVNGLKKKLRDSMETFNRLREVISSEYRETVQRRYFTVTGENPDDKTLDLLISSGESETFLQKAIQEQ-GRGRIIDTINEIQERHDAVKELEKNLLALHQVFLDMTVLVQFQ 245
L. culinaris GFBR01017428.1 121 GCGPGSSSDRTRSSVVNGLKKKLRDSMETFNRLREVISSEYRETVQRRYFTVTGENPDDKTLDLLISSGESETFLQKAIQEQ-GRGRIIDTINEIQERHDAVKELEKNLLALHQVFLDMTVLVQFQ 245
L. odemensis 121 GCGPGSSSDRTRSSVVNGLKKKLRDSMETFNRLREVISSEYRETVQRRYFTVTGENPDDKTLDLLISSGESETFLQKAIQEQ-GRGRIIDTINEIQERHDAVKELEKNLLALHQVFLDMTVLVQFQ 245
H. vulgare Ror2 AY246907.1 247 GEQLDDIEGHVGRARSFVDRGREQLQVARKHQKSSRKWTFIGIGILLVVILIIVIPIVLKNTNKSNNNN----------SQQ-------------------- 318
A. thaliana AEE75103.1 syntaxin-121 249 GAQLDDIESHVGRASSFIRGGTDQLQTARVYQKNTRKWTCIAIIILIIIITVVVLAVLKPWNNSSGGGGGGGGGTTGGSQPN-SGTPPNPPQARRLLR- 346
A. ipaensis XP 019428840.1 247 GEQLDDIESHVARAHSFVHTGTERLQTARKHQKNTRKWTCFCIILLAIILVVLLVVLKPWQHNGSSG---------GGSQPAPAQTPPPPPPAGA---- 333
C. cajan XP_020240337.1 242 GEQLDDIESHVARANSYVRGGVQQLHVARKHQKNTRKWTCIAIILLIIIILVIVLPIVLRN--- 302
C. cajan XP_020220797.1 245 GEQLDDIESHVARAHSFVRTGAEQLQTARKHQKNTRKWTCYCIILLVIILLVVLFTVRPWKHSSSGGS--------DGSQPAPAQTPPPPPVNA----- 331
P. vulgaris XP_007145783. 247 GEQLDDIESHVARAQSFVHTGTEQLQTARKHQKNTRKWTCYCIILLVIILVVVLSIVRPWEGSSNSGN--------GGNQSTPAQTPPSPPPPAQP--- 335
G. max XP_003519112.1 247 GEQLDDIESHVARAHSFVRTGAEQLQTARKHQKNTRKWTCYCIILLVIILFVVLFTVKPWENSSSGGN--------GGGQPAPAQTPPSPSPPVNA--- 335
L. angustifolius XP_019428840.1 245 GEQLNDIESHVARAHSFVQTGVEQLHTARKHQRSTRKWTCYAIILVIVLFVVLFTVRPWEKKGSSGN--------GGNQPASAQTPPLSPPPPTA--- 333
C. arietinum XP_004497646.1 246 GEQLDNIENHVARASSFVHNGAEQLQTARKHQKNTRKWTCYCIILILIIVLLVVLFTVKPWKNSDSGGS--------GGSQPAPAQTTPSPPPPA----- 332
M. truncatula XP_003590328.1 246 GEQLDNIESHVARANSYVRGGVQQLHVARKHQMNTRKWTCIAIILLIIILIIVLPIVLKK-- 306
M. truncatula XP_003590329.1 syntaxin 122 protein 250 GEQLDDIESHVARASSFVHTGTDQLQTARKHQKNTRKWACYCIILLIIVLIVVLATVKPWQHNDSGGG--------GGNQPAPAQTPPSPPPPSGGT-- 339
L. culinaris cv. Alpo 1 246 GEQLDDIESHVARASSFVHTGAEHLETARKHQRNTRKWTCYLIILILIIVLVVVLIVVKPWQHD--GGG--------GDSQPAPAQTTPSPPPPSPPAST 335
L. culinaris cv. Alpo 2 246 GEQLDDIESHVARASSFVHTGAEHLETARKHQRNTRKWTCYLIILILIIVLVVVLIVVKPWQHD--GGG--------GDSQPAPAQTTPSPPPPSPPAST 335
L. culinaris GFBR01017428.1 246 GEQLDDIESHVARASSFVHTGAEHLETARKHQRNTRKWTCYLIILILIIVLVVVLIVVKPWQHD--GGG--------GDSQPAPAQTTPSPPPPSPPAST 335
L. odemensis 246 GEQLDDIESHVARASSFVHTGAEHLETARKHQRNTRKWTCYLIILILIIVLVVVLIVVKPWQHD--GGG--------GDSQPAPAQTTPSPPPPSPPAST 335
