# Supplementary material for: A genome-wide identification and comparative analysis of the lentil MLO genes
Source: PLoS One. 2018 Mar 23;13(3):e0194945. doi: 10.1371/journal.pone.0194945 (PMC5865747; doi:10.1371/journal.pone.0194945)
Supplement: S1 Table — (PDF) [file pone.0194945.s006.pdf]

Supplementary Table S1. Online prediction servers and tools used for the analysis of lentil *MLO* gene family

Resources	**Characteristics and purpose**	**Web address**	**References**
SignalP 4.1	Prediction of signal peptide	http://www.cbs.dtu.dk/services/SignalP	41
TMHMM v2.0	Prediction of transmembrane domains	http://www.cbs.dtu.dk/services/TMHMM/	42
CCTOP	Prediction of transmembrane domains	http://cctop.enzim.ttk.mta.hu/	43
Plant-mPloc	Prediction of protein subcellular localisation	http://www.csbio.sjtu.edu.cn/bioinf/plant-multi/	44
InterProScan	Protein domain identification	http://www.ebi.ac.uk/interpro/search/sequence-search	45
CaM	Prediction of calmodulin binding domains	http://cam.umassmed.edu/Seqsearch.php	46
ExPASy	Compute pI/Mw tool	http://web.expasy.org/compute_pi/	47
