# Supplementary material for: A genome-wide identification and comparative analysis of the lentil MLO genes
Source: PLoS One. 2018 Mar 23;13(3):e0194945. doi: 10.1371/journal.pone.0194945 (PMC5865747; doi:10.1371/journal.pone.0194945)
Supplement: S2 Table — (PDF) [file pone.0194945.s007.pdf]

Supplementary Table S2.-Analyzed accessions of cultivated and wild relatives of lentil.

**Accession ID**	**Species**	**Country of origin**	**Source**
[S] Alpo BGE025606	*Lens culinaris* subsp. *culinaris*	Spain	Univ. of León (Spain)
[S] Lupa BGE008693	*Lens culinaris* subsp. *culinaris*	Spain	Univ. of León (Spain)
Tetir BGE016363	*Lens culinaris* subsp. *culinaris*	Canary I. (Spain)	CRF-INIA (Spain)
Mala BGE029091	*Lens culinaris* subsp. *culinaris*	Canary I. (Spain)	CRF-INIA (Spain)
BGE016364	*Lens culinaris* subsp. *culinaris*	Canary I. (Spain)	CRF-INIA (Spain)
BGE016365	*Lens culinaris* subsp. *culinaris*	Canary I. (Spain)	CRF-INIA (Spain)
Verdina	*Lens culinaris* subsp. *culinaris*	Spain	Univ. of León (Spain)
WA8649041	*Lens culinaris* subsp. *culinaris*	USA	Washington State Univ.
WA8649090	*Lens culinaris* subsp. *culinaris*	USA	Washington State Univ.
ILL52	*Lens culinaris* subsp. *culinaris*	Iraq	ICARDA
ILL60	*Lens culinaris* subsp. *culinaris*	Iraq	ICARDA
ILL62	*Lens culinaris* subsp. *culinaris*	Iraq	ICARDA
ILL69	*Lens culinaris* subsp. *culinaris*	Cyprus	ICARDA
ILL74	*Lens culinaris* subsp. *culinaris*	Chile	ICARDA
ILL77	*Lens culinaris* subsp. *culinaris*	Chile	ICARDA
ILL92	*Lens culinaris* subsp. *culinaris*	Ukraine	ICARDA
ILL96	*Lens culinaris* subsp. *culinaris*	Morocco	ICARDA
ILL144	*Lens culinaris* subsp. *culinaris*	Turkey	ICARDA
ILL191	*Lens culinaris* subsp. *culinaris*	Lebanon	ICARDA
ILL296	*Lens culinaris* subsp. *culinaris*	Greece	ICARDA
ILL323	*Lens culinaris* subsp. *culinaris*	Serbia	ICARDA
ILL343	*Lens culinaris* subsp. *culinaris*	Italy	ICARDA
ILL389	*Lens culinaris* subsp. *culinaris*	Chile	ICARDA
ILL442	*Lens culinaris* subsp. *culinaris*	Chile	ICARDA
ILL458	*Lens culinaris* subsp. *culinaris*	Chile	ICARDA
ILL459	*Lens culinaris* subsp. *culinaris*	Chile	ICARDA
ILL508	*Lens culinaris* subsp. *culinaris*	Italy	ICARDA
ILL526	*Lens culinaris* subsp. *culinaris*	Iran	ICARDA
ILL590	*Lens culinaris* subsp. *culinaris*	Turkey	ICARDA
ILL662	*Lens culinaris* subsp. *culinaris*	Turkey	ICARDA
ILL868	*Lens culinaris* subsp. *culinaris*	Iran	ICARDA
ILL913	*Lens culinaris* subsp. *culinaris*	Spain	ICARDA
ILL918	*Lens culinaris* subsp. *culinaris*	Tunisia	ICARDA
ILL1021	*Lens culinaris* subsp. *culinaris*	Iran	ICARDA
ILL1199	*Lens culinaris* subsp. *culinaris*	Iran	ICARDA
ILL1323	*Lens culinaris* subsp. *culinaris*	Iran	ICARDA
ILL1349	*Lens culinaris* subsp. *culinaris*	Iran	ICARDA
ILL1370	*Lens culinaris* subsp. *culinaris*	Iran	ICARDA
ILL1645	*Lens culinaris* subsp. *culinaris*	Colombia	ICARDA
ILL1827	*Lens culinaris* subsp. *culinaris*	Chile	ICARDA
ILL1832	*Lens culinaris* subsp. *culinaris*	Chile	ICARDA
ILL1840	*Lens culinaris* subsp. *culinaris*	Chile	ICARDA
ILL1841	*Lens culinaris* subsp. *culinaris*	Chile	ICARDA
ILL1842	*Lens culinaris* subsp. *culinaris*	Chile	ICARDA
ILL1845	*Lens culinaris* subsp. *culinaris*	Chile	ICARDA
ILL1879	*Lens culinaris* subsp. *culinaris*	Turkey	ICARDA

ILL1890	*Lens culinaris* subsp. *culinaris*	Tunisia	ICARDA
ILL1918	*Lens culinaris* subsp. *culinaris*	Austria	ICARDA
ILL2249	*Lens culinaris* subsp. *culinaris*	Iran	ICARDA
ILL2257	*Lens culinaris* subsp. *culinaris*	Iran	ICARDA
ILL2307	*Lens culinaris* subsp. *culinaris*	Chile	ICARDA
ILL3042	*Lens culinaris* subsp. *culinaris*	India	ICARDA
ILL3286	*Lens culinaris* subsp. *culinaris*	India	ICARDA
ILL3312	*Lens culinaris* subsp. *culinaris*	India	ICARDA
ILL3467	*Lens culinaris* subsp. *culinaris*	Ethiopia	ICARDA
ILL3510	*Lens culinaris* subsp. *culinaris*	India	ICARDA
ILL3596	*Lens culinaris* subsp. *culinaris*	India	ICARDA
ILL5588	*Lens culinaris* subsp. *culinaris*	Jordan	ICARDA
[S] BGE016880	*Lens culinaris* subsp. *orientalis*	Israel	CRF-INIA (Spain)
ILWL7	*Lens culinaris* subsp. *orientalis*	Turkey	ICARDA
ILWL11	*Lens culinaris* subsp. *orientalis*	Syria	ICARDA
LENS166/92	*Lens culinaris* subsp. *orientalis*	Unknown	Dr. Rubiales (Spain)
[S] ILWL235	*Lens odemensis*	Syria	ICARDA
[S] ILWL39	*Lens odemensis*	Turkey	ICARDA
ILWL21	*Lens odemensis*	Israel	ICARDA
ILWL164	*Lens odemensis*	Syria	ICARDA
ILWL238	*Lens odemensis*	Syria	ICARDA
ILWL252	*Lens odemensis*	Syria	ICARDA
[S] 244	*Lens lamottei*	Morocco	Prof. G. Ladizinsky (Israel)
ILWL29	*Lens lamottei*	Spain	ICARDA
ILWL428	*Lens lamottei*	Spain	ICARDA
[S] BGE016873	*Lens nigricans*	Fr. Yugoslavia	CRF-INIA (Spain)
BGE019580	*Lens nigricans*	Spain	CRF-INIA (Spain)
BGE019582	*Lens nigricans*	Spain	CRF-INIA (Spain)
ILWL30	*Lens nigricans*	Spain	ICARDA
[S] 133	*Lens tomentosus*	Turkey	Prof. G. Ladizinsky
ILWL90	*Lens tomentosus*	Turkey	ICARDA
ILWL120	*Lens tomentosus*	Syria	ICARDA
[S] BGE016877	*Lens ervoides*	Israel	CRF-INIA (Spain)
ILWL43	*Lens ervoides*	Croatia	ICARDA
ILWL54	*Lens ervoides*	Israel	ICARDA

[S] *MLO1* and *MLO3* complete sequences obtained in this work (Fig 6).
