# Supplementary material for: A genome-wide identification and comparative analysis of the lentil MLO genes
Source: PLoS One. 2018 Mar 23;13(3):e0194945. doi: 10.1371/journal.pone.0194945 (PMC5865747; doi:10.1371/journal.pone.0194945)
Supplement: S3 Table — (PDF) [file pone.0194945.s008.pdf]

Supplementary Table 3. Sets of primers used for amplification of *MLO1* and *MLO3* genes.

Gene and primer pair	Primer name	Sequence (5'-3')	Amplicon size (bp)*
MLO1 / A	Pro0028-F<br>03E0870-R	GCCCTTCAATATTTCATGACC<br>CAAGAATTCTCCGAGGTATTGG	843
MLO1 / A2	01E0230-F<br>04E1066-R	TTTGGAAGAGACGCCAACTT<br>GTTTAACGGCCACAATACGC	837
MLO1 / A3	Pro0160-F<br>03E0852-R	AATCATCTCAAGTTGCAACAAA<br>TTGGATCAAAATACTCCAAGAG	693
MLO1 / B	03E0747-F<br>06E1748-R	ACTTGGCATCCTTGTTCCAC<br>GTTGTGTCCCTTGCAAACCT	1002
MLO1 / C	05E1356-F<br>09E2479-R	ATGCGGAAGTGGAAGACTTG<br>AAAGCACTGTGAAAAGCCAGA	1124
MLO1 / C2	05E1356-F<br>08E2187-R	ATGCGGAAGTGGAAGACTTG<br>TTCCTACTACAACTTTAAAATCCTCTT	832
MLO1 / D	08E2117-F<br>11E2862-R	TGATGCACAATTTGATTTCCA<br>AGAAGAAGGCGAGGACGATT	746
MLO1 / E	11E2732-F<br>14E3440-R	GGTGCTAAGTTACAAATGATCATAAC<br>AGCATAAAGAGGCAAAGTCACA	709
MLO1 / F	13E3254-F<br>15E4165-R	TTGCTTCCACAAAACCACTG<br>GTTGATGGCTGGCTTGAGTT	912
MLO1 / F2	14E3419-F<br>15E4235-R	TGTGACTTTGCCTCTTTATGCT<br>TTGTGGACTGTCGCTGTTTC	817
MLO1 / G	15E4026-F<br>Ter4541-R	ATGGGATCAACCATGAAACC<br>TCTTCATCTGCAAGAGTGTACC	516
MLO1 / G2	15E4026-F<br>15E4457-R	ATGGGATCAACCATGAAACC<br>TTCTAATCGCTCCCTCCCTA	432
MLO3 / A-M3	Pro0071-F<br>03E0919-R	GCGTCAAAGTTCAACAGCTT<br>GCTGGTGAATTCCATCTGCT	849
MLO3 / A2-M3	01E0323-F<br>03E0919-R	TGGAGAAGGTGGAGGAAGAA<br>GCTGGTGAATTCCATCTGCT	597
MLO3 / B-M3	03E0769-F<br>06E1465-R	AATGCCGCTAAAGGTGAAGA<br>TGACCTTACAAATTGCCTGAA	697
MLO3 / C-M3	05E1295-F<br>10E2130-R	TGGAAGAAGACACTTGAGTTTTTG<br>CTTGCAACTTTGTTCCAACC	836
MLO3 / C2-M3	07E1626-F<br>11E2411-R	CATTTGGCACCTTCAAGTCA<br>GCAGTCCATGAAAAGAAAGCA	786
MLO3 / D-M3	09E1981-F<br>14E2948-R	TCTCTGGCTACCATTTGTTCC<br>CCCACGGTTCTGCTTTATGT	968
MLO3 / E-M3	13E2723-F<br>Ter3311-R	TGCAGCTATGTGACTCTTCCTC<br>ACAAACCAATCATCATCATCACA	589
MLO3 / E2-M3	13E2723-F<br>14E3282-R	TGCAGCTATGTGACTCTTCCTC<br>CCAATTGATTTGTGGCCATT	560

* Expected size in *Lens culinaris subsp. culinaris* cv. CDC-Redberry
