# Supplementary material for: A genome-wide identification and comparative analysis of the lentil MLO genes
Source: PLoS One. 2018 Mar 23;13(3):e0194945. doi: 10.1371/journal.pone.0194945 (PMC5865747; doi:10.1371/journal.pone.0194945)
Supplement: S4 Table — (DOCX) [file pone.0194945.s009.docx]

**Supplementary Table S4**. Characteristics of full length lentil *MLO* genes

| Gene name | Chromosome | Gene number  pre-release | Number of exons | Strand | Length (bp/aa) | | | Protein characteristics | |
| --- | --- | --- | --- | --- | --- | --- | --- | --- | --- |
|  |  |  |  |  | Genomic | CDS | Protein | pI | kDa |
| *LcMLO1* | LcChr2 | Lc09371 | 15 | + | 4263 | 1734 | 577 | 9.25 | 66.36 |
| *LcMLO2* | LcChr5 | Lc20676 | 15 | - | 3231 | 1617 | 538 | 8.73 | 62.40 |
| *LcMLO3* | LcChr2 | Lc10247 | 14 | - | 2970 | 1710 | 569 | 9,17 | 65.07 |
| *LcMLO4* | LcChr2 | Lc09186 | 14 | + | 3272 | 1464 | 487 | 7.01 | 56.63 |
| *LcMLO5* | LcChr3 | Lc13589 | 15 | - | 3750 | 1635 | 544 | 6.80 | 61,50 |
| *LcMLO6* | LcChr5 | Lc23149 | 14 | + | 4542 | 1740 | 579 | 9.06 | 66-68 |
| *LcMLO7* | LcChr2 | Lc10256 | 15 | + | 3332 | 1662 | 553 | 8.88 | 63.89 |
| *LcMLO8* | LcChr3 | Lc10926 | 13 | + | 2907 | 1662 | 553 | 9.14 | 63.80 |
| *LcMLO9* | LcChr3 | Lc13662 | 13 | + | 4431 | 1638 | 545 | 8.90 | 61.43 |
| *LcMLO10A* | LcContig747647 | Lc38195 | 15 | + | 3627 | 1797 | 598 | 9.17 | 68.67 |
| *LcMLO10B* | LcChr5 | Lc20048 | 15 | - | 4106 | 1734 | 577 | 8.84 | 65.50 |
| *LcMLO11* | LcChr4 | Lc18408 | 13 | - | 9816 | 1647 | 548 | 6.45 | 62.83 |
| *LcMLO13* | LcChr7 | Lc29426 | 13 | - | 3747 | 1566 | 521 | 6.77 | 59.66 |
| *LcMLO14* | LcChr7 | Lc29427 | 13 | - | 2949 | 1569 | 522 | 8.94 | 58.94 |
| *LcMLO15* | LcChr2 | Lc06042 | 15 | + | 5262 | 1653 | 550 | 9.40 | 62.85 |
