# Supplementary material for: A genome-wide identification and comparative analysis of the lentil MLO genes
Source: PLoS One. 2018 Mar 23;13(3):e0194945. doi: 10.1371/journal.pone.0194945 (PMC5865747; doi:10.1371/journal.pone.0194945)
Supplement: S5 Table — (PDF) [file pone.0194945.s010.pdf]

Supplementary Table S5. Protein characteristics and identity score of lentil MLOs with its closest homologues in *M. truncatula* and *A. thaliana*.

Gene name	Interproscan prediction		Subcellular localization[a]	SP[b]	Trasmembane domains[c]			Identity with *M. truncatula*					Identity with *A. thaliana*				
	Pfam Ref.	Name	Plant_mPloc		T	I	CCTOP	*M. truncatula* gene name	Blastp hit		Pair-wise (%)		*A. thaliana* gene name	Blastp hit		Pair-wise (%)	
									ID	E-value	Id	Sim		ID	E-value	Id	Sim
LcMlO1	PF03094	MLO-related	CM	NO	7	7	7	*Medtr6g033330*	84	0.0	83.4	90.2	*ATMLO6*	61	0.0	60.2	74.4
LcMlO2	PF03094	MLO-related	CM	NO	7	7	7	*Medtr5g022400*	85	0.0	81.3	91.4	*ATMLO4*	48	4e-^{156}	44.2	59.6
LcMlO3	PF03094	MLO-related	CM	NO	7	7	7	*Medtr2g093750*	80	0.0	80.1	86.2	*ATMLO6*	62	0.0	59.1	72.2
LcMlO4	PF03094	MLO-related	CM	NO	7	7	7	*Medtr2g028470*	85	0.0	83.5	90.0	*ATMLO13*	57	1e-^{172}	52.3	66.0
LcMlO5	PF03094	MLO-related	CM	NO	7	7	7	*Medtr3g115960*	85	0.0	84.2	88.5	*ATMLO5*	57	0.0	56.1	69.7
LcMlO6	PF03094	MLO-related	CM	NO	8	8	8	*Medtr5g024700*	80	0.0	78.9	86.2	*ATMLO8*	58	0.0	55.5	67.4
LcMlO7	PF03094	MLO-related	CM	NO	7	7	7	*Medtr2g006280*	91	0.0	91.3	94.9	*ATMLO4*	65	0.0	65.0	77.9
LcMlO8	PF03094	MLO-related	CM	NO	7	7	7	*Medtr3g115940*	90	0.0	90.1	94.6	*ATMLO12*	49	0.0	47.5	64.0
LcMlO9	PF03094	MLO-related	CM	NO	7	7	7	*Medtr3g118190*	84	0.0	83.8	87.8	*ATMLO1*	70	0.0	64.8	72.8
LcMlO10A	PF03094	MLO-related	CM	NO	8	7	8	*Medtr8g073130*	75	0.0	75.0	84.7	*ATMLO8*	58	0.0	56.0	70.8
LcMlO10B	PF03094	MLO-related	CM	NO	7	7	7	*Medtr8g073130*	80	0.0	76.7	84.4	*ATMLO8*	63	0.0	56.7	69.4
LcMlO11	PF03094	MLO-related	CM	NO	7	7	7	*Medtr4g079140*	82	0.0	80.4	86.5	*ATMLO12*	42	2e-^{151}	38.8	57.4
LcMLO13	PF03094	MLO-related	CM	NO	7	7	7	*Medtr8g010500*	65	0.0	60.5	74.2	*ATMLO1*	63	0.0	57.5	67.7
LcMlO14	PF03094	MLO-related	CM	NO	7	7	7	*Medtr8g010530*	81	0.0	80.5	88.5	*ATMLO1*	67	0.0	62.0	72.8
LcMlO15	PF03094	MLO-related	CM	NO	7	7	7	*Medtr2g079500*	92	0.0	91.7	96.0	*ATMLO11*	69	0.0	68.1	77.9

[a] Subcellular localization as predicted by the web server Plant-mPloc. CM stand for cell membrane

[b] Prediction of Signal peptide (SP) as indicated by SignalP server.

[c] Number of transmembrane domains (TM) as predicted by TMHMM (T) Interproscan5 (I) and CCTOP web servers
