# Supplementary material for: A genome-wide identification and comparative analysis of the lentil MLO genes
Source: PLoS One. 2018 Mar 23;13(3):e0194945. doi: 10.1371/journal.pone.0194945 (PMC5865747; doi:10.1371/journal.pone.0194945)
Supplement: S6 Table — (PDF) [file pone.0194945.s011.pdf]

Supplementary Table S6. Conserved amino acids in MLO sequences according Elliot et al., 2005 [45]

Elliot et al. 2005		Number of lentil MLOs in which the residue is present and alternatives		Elliot et al. 2005		Number of lentil MLOs in which the residue is present and alternatives	
Amino acid	Position			Amino acid	Position		
E	35	13	H, V	F	227	15	-
M	65	15	-	F	240	15	-
G	68	15		Y	243	14	F
S	71	15	-	W	263	15	-
L	74	14	M	P	287	15	-
C	86	15	-	F	329	15	-
C	98	15	-	W	330	15	-
C	114	15	-	P	334	15	-
F	135	12	S	F	346	14	I
W	158	15	-	N	348	14	I
E	163	15	-	F	350	14	Y
F	207	15	-	C	367	15	-
Q	210	15	-	T	393	14	-
Y	220	15	-	P	395	14	-
R	224	15	-	W	423	15	H
